# APF-YOLOV8: Enhancing Multiscale Detection and Intra-Class Variance Handling for UAV-Based Insulator Power Line Inspections

**DOI:** 10.12688/f1000research.160650.2

**Published:** 2025-06-23

**Authors:** Rita Aitelhaj, Badr-Eddine Benelmostafa, Hicham Medromi

**Affiliations:** 1 System Architecture Team (EAS), Engineering Research Laboratory (LRI),, National High School of Electricity and Mechanic (ENSEM), Hassan II University, Casablanca, Morocco, CASABLANCA, Morocco

**Keywords:** UAV Power Line Inspection, Automated Insulator identification, Real time Object detection algorithms, Improved YOLOV8, Multi-scale Object size Challenge, Intra-Class Invariance, Computer Vision

## Abstract

**Background:**

UAV-based power line inspections offer a safer, more efficient alternative to traditional methods, but insulator detection presents key challenges: multiscale object detection and intra-class variance. Insulators vary in size due to UAV altitude and perspective changes, while their visual similarities across types (e.g., glass, porcelain, composite) complicate classification.

**Methods:**

To address these issues, we introduce APF-YOLO, an enhanced YOLOv8-based model integrating the Adaptive Path Fusion (APF) neck and the Adaptive Feature Alignment Module (AFAM). AFAM balances fine-grained detail extraction for small objects with semantic context for larger ones through local and global pathways by integrating advanced attention mechanisms. This work also introduces the Merged Public Insulator Dataset (MPID), a comprehensive dataset designed for insulator detection, representing diverse real-world conditions such as occlusions, varying scales, and environmental challenges.

**Results:**

Evaluations on MPID demonstrate that APF-YOLO surpasses state-of-the-art models with different neck configurations, achieving at least a +2.71% improvement in mAP@0.5:0.9 and a +1.24% increase in recall, while maintaining real-time performance in server-grade environments. Although APF-YOLO adds computational requirements, these remain within acceptable limits for real-world applications. Future work will optimize APF-YOLO for edge devices through techniques such as model pruning and lightweight feature extractors, enhancing its adaptability and efficiency.

**Conclusion:**

Combined with MPID, APF-YOLO establishes a strong foundation for advancing UAV-based insulator detection, contributing to safer and more effective power line monitoring.

## Introduction

The rapid integration of drones and artificial intelligence (AI) is transforming the maintenance of electrical infrastructure, enabling more efficient inspection and monitoring processes. ^1^^–^^7^ Among critical components, insulators play a pivotal role in ensuring the safety and reliability of electrical systems. ^8^ Failure to detect anomalies in these components can result in costly repairs, unplanned outages, and potential hazards to personnel and the public. By combining aerial surveillance with AI-driven analytics, utilities can identify and address issues proactively, reducing downtime and enhancing system performance to ensure safer and more reliable energy delivery. ^1^^,^^2^

**Figure 1.**
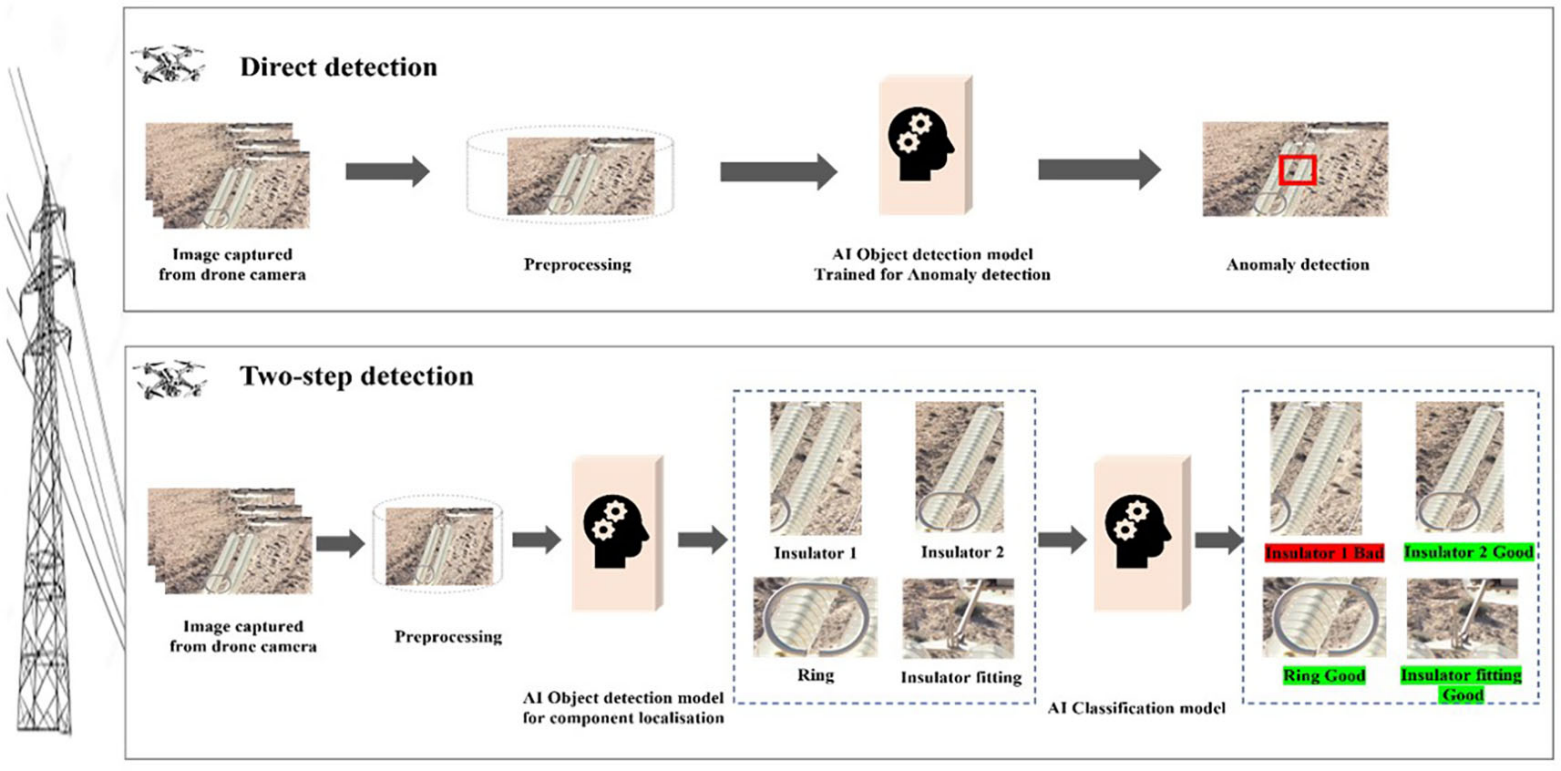
General comparative framework for direct vs. two-step object detection in insulator anomaly classification.

Current methodologies for anomaly detection in insulators can be broadly classified into two approaches: direct detection and two-step detection. As shown in Figure 1, direct detection aims for immediate identification of anomalies and is valued for its simplicity and speed. However, its high false positive rate often necessitates additional verification, limiting its reliability in high-stakes scenarios. Conversely, the two-step approach, which involves localizing power line objects before classifying them, offers greater precision and reliability. The second step introduces a computational trade-off, requiring additional processing to achieve its enhanced accuracy. ^3^^,^^4^ Our research focuses on the initial phase of this two-step methodology, specifically targeting the accurate localization of insulator to streamline subsequent classification processes.

Building on this foundation, AI object detection algorithms, such as YOLO (You Only Look Once), ^9^ have demonstrated exceptional performance in real-time detection. Their ability to process visual data rapidly makes them highly suitable for drone-based inspections. ^10^^,^^11^ However, several challenges hinder their effectiveness in electrical inspections, including intra-class variance and multi-scale detection. Intra-class variance arises from differences among insulators of the same type, such as variations caused by wear, environmental conditions, or manufacturing differences. Multi-scale detection presents additional challenges due to the significant variation in insulator sizes captured in drone imagery. Wide-angle shots often lack the resolution required for detailed analysis, while close-ups demand slow and precise drone operation, complicating the inspection process. ^12^ Addressing these issues is crucial to improving the reliability and accuracy of AI-driven inspections. ^5^^,^^6^

To tackle these challenges, we propose an enhanced YOLOv8 model called Adaptive Path Fusion YOLO (APF-YOLO) that optimizes the detection process by refining its neck architecture for effective scale fusion. This enhancement enables the model to better handle the diverse range of insulator sizes encountered during inspections, improving detection precision while maintaining real-time performance. By reducing false positives and accommodating intra-class variability, our approach offers a more reliable solution for insulator localization. Future extensions of this work will focus on robust classification techniques to complement the localization process, facilitating a comprehensive and scalable solution for anomaly detection in electrical infrastructure. Through these contributions, we aim to advance the field of drone-based electrical inspections, promoting safer and more efficient maintenance practices. ^7^

## Related works

Object detection has advanced significantly in recent years, driven by the development of deep learning-based architectures. Despite this progress, challenges such as small object detection, multi-scale feature management, and intra-class variance handling remain areas where further improvements are required, especially in specialized applications like UAV-based power line inspections. ^12, 13^ In these scenarios, the varying scales of objects due to UAV altitude and perspective shifts, as well as the visual similarities across different insulator types, create additional complexities. While frameworks such as Faster R-CNN, RT-DETR, and others have been developed to address object detection, this study focuses exclusively on state-of-the-art (SOTA) YOLO-based models, particularly their neck configurations. Non-YOLO architectures are not considered, as the primary contribution lies in advancing YOLO neck designs. This section reviews recent developments in YOLO-based models and positions APF-YOLO within this landscape.

### Multi-scale feature management

Effectively managing multi-scale features is a cornerstone of robust object detection, enabling models to handle objects of varying sizes and resolutions. YOLO-based models have tackled this challenge through diverse architectural innovations and feature integration strategies.

The YOLOv8-baseline model ^14^ lays the groundwork by incorporating a Pyramid Attention Network-Feature Pyramid Network (PAN-FPN), ^15, 16^ which facilitates the fusion of high-level semantic features with low-level spatial details. This combination ensures robust detection of objects at multiple scales. Complementing this, the integration of the Cross-Stage Partial Network (CSPNet) ^17^ optimizes feature reuse, making multi-scale feature extraction both efficient and effective.

Building on this solid foundation, YOLOv8-P2 introduces an additional P2 layer that significantly enhances granularity, particularly for small-object detection. This improvement proves invaluable in environments with dynamic object scales, such as those encountered in UAV-based applications. Further extending this lineage, YOLOv9 ^18^ incorporates the Generalized Efficient Layer Aggregation Network (GELAN), an advanced architecture designed to minimize information loss during forward propagation. GELAN’s ability to aggregate features without introducing bottlenecks allows for precise detection of both small and large objects, a capability further enhanced by the use of Programmable Gradient Information (PGI) to ensure robust training.

While these models focus on general-purpose enhancements, other YOLO variants target specific challenges in multi-scale detection. For instance, YOLOv8-MAFPN ^19^ introduces the Multi-Auxiliary Feature Pyramid Network, which leverages a dual fusion strategy: the Superficial Assisted Fusion (SAF) module integrates shallow features for preserving small-object details, while the Advanced Assisted Fusion (AAF) module conveys diverse gradient information for deeper feature learning. Similarly, YOLOv8-HSFPN ^20^ employs a High-level Screening Feature Pyramid (HS-FPN), dynamically filtering and fusing features to achieve a balanced representation. This approach is particularly effective in complex scenarios like medical imaging and industrial inspection.

Another noteworthy model, YOLOv8-bifpn, ^21^ incorporates a Bi-Directional Feature Pyramid Network (BiFPN), which allows for seamless top-down and bottom-up feature flow. This bidirectional connectivity, coupled with weighted fusion, enables efficient handling of multi-scale features, making it suitable for applications requiring both speed and accuracy.

Attention mechanisms also play a critical role in enhancing multi-scale capabilities. For example, YOLOv8-ASF-P2 ^22^ integrates Attentional Scale Sequence Fusion, which prioritizes features based on their relevance at different scales. Initially developed for medical imaging, this method has shown promise in detecting small and dense objects in other challenging domains, such as aerial imagery. Likewise, YOLOv8-CFPT-P2345 ^23^ introduces the Cross-Layer Feature Pyramid Transformer (CFPT), which avoids traditional semantic gaps by leveraging attention blocks for cross-layer global information fusion. This design is particularly advantageous for detecting small objects where detail preservation is critical.

Finally, YOLOv10 ^24^ combines these advancements into a holistic framework. Its efficiency-accuracy-driven design incorporates lightweight classification heads, spatial-channel decoupled downsampling, and rank-guided blocks. These features collectively enable high-performance multi-scale detection with reduced computational overhead, making it ideal for real-world, resource-constrained applications.

### Intra-class variance handling

Addressing intra-class variance—where objects of the same class exhibit significant differences in appearance due to material, texture, or environmental conditions—is another critical challenge in object detection. YOLO-based models have approached this problem with a variety of innovative techniques.

The YOLOv8-baseline ^14^ model addresses intra-class variance through its anchor-free design, which allows for greater flexibility in adapting to variations in object shape and size. Building on this, YOLOv8-P2 leverages its P2 layer to extract fine-grained features, making it particularly adept at differentiating visually similar objects within the same class.

A more advanced solution comes from YOLOv9, which introduces Programmable Gradient Information (PGI) to focus the model’s attention on distinguishing features. By ensuring reliable gradient flow and minimizing information loss, PGI enables robust handling of intra-class variance, even in dynamic environments. Complementing this, the GELAN architecture efficiently aggregates features while preserving subtle details, enhancing the model’s ability to differentiate between closely related objects.

For scenarios requiring a more nuanced approach, YOLOv8-MAFPN aligns features across scales to capture subtle variations within the same class. Similarly, YOLOv8-HSFPN combines shallow and deep feature extraction through its HS-FPN module, achieving better representation in domains like medical imaging, where intra-class variance is particularly pronounced.

Other YOLO variants incorporate specialized mechanisms to tackle this issue. YOLOv8-goldyolo, ^25^ for example, employs a Gather-and-Distribute (GD) mechanism that aggregates features to enhance differentiation. It further introduces MAE-style pretraining, an innovative approach that leverages unsupervised learning to improve robustness. Likewise, YOLOv8-ASF-P2 integrates Channel and Position Attention Mechanisms (CPAM) to emphasize the most discriminative features, demonstrating superior performance in detecting small and densely packed objects, such as in cell segmentation.

Finally, YOLOv10 takes a step forward with its consistent dual assignments for NMS-free training. By aligning supervisory signals, this approach improves feature extraction and strengthens the model’s ability to handle intra-class variance across diverse conditions. Coupled with its efficiency-driven design, YOLOv10 delivers robust performance while maintaining low computational costs, cementing its place as a cutting-edge solution for object detection.

### Our contribution

In this landscape, APF-YOLO introduces a novel neck configuration to address key challenges in multi-scale feature management and intra-class variance handling, particularly for UAV-based power line inspections. The proposed Adaptive Path Fusion (APF) neck synthesizes advancements from previous YOLO-based models, offering seamless integration of multi-scale features while maintaining computational efficiency. Additionally, the Adaptive Feature Alignment Module (AFAM) ensures robust alignment of features across scales, enabling accurate detection of objects with diverse sizes and characteristics. APF-YOLO integrates advanced attention mechanisms, including Efficient Local Attention (ELA), Efficient Channel Attention (ECA), and Context Anchor Attention (CAA). These mechanisms amplify the model’s ability to detect and differentiate objects under varying environmental and geometric conditions. Collectively, these innovations position APF-YOLO as a significant advancement in UAV-based detection, particularly in addressing the nuanced challenges of insulator detection.

To provide a comprehensive overview of the advancements in YOLO-based neck configurations and their approaches to addressing multi-scale object detection and intra-class variance handling, Table 1 summarizes the key contributions and release years of the reviewed models. This table highlights the evolutionary trajectory of these architectures and situates APF-YOLO within the broader landscape of YOLO-based innovations.

**Table 1.** Comparative analysis of YOLO based models: Addressing small object detection, multi-scale feature fusion, and intra-class variance.

Model/approach	Challenge: Multi-scale object detection	Challenge: Intra-class variance handling	Context/application	Year of release
YOLOv8-baseline ^14^	Uses Feature Pyramid Networks (FPN) to merge semantic and spatial features across resolutions. Incorporates Cross-Stage Partial Network (CSPNet) for efficient multi-scale feature reuse.	Adopts an anchor-free detection approach to improve flexibility in detecting objects of varying shapes and sizes.	General-purpose object detection (real-time applications).	2023
YOLOv8-P2 ^14^	Introduces an additional P2 layer in the FPN, enhancing small-object detection by improving granularity of features for lower-resolution scales.	Leverages the P2 layer to extract fine-grained features, facilitating differentiation of visually similar small objects.	General-purpose object detection (real-time applications).	2023
YOLOv9 ^18^	Employs Generalized Efficient Layer Aggregation Network (GELAN) for optimized multi-scale feature fusion with minimal information loss during forward propagation.	Utilizes Programmable Gradient Information (PGI) to guide training, emphasizing class-distinctive features and improving robustness in intra-class variance.	General-purpose object detection.	2024
YOLOv8-MAFPN ^19^	Uses Multi-Auxiliary Feature Pyramid Networks (MAFPN) to integrate shallow and deep feature layers, ensuring balanced feature representations across scales.	Aligns features across scales using Superficial and Advanced Assisted Fusion (SAF and AAF) to capture subtle object differences effectively.	General object detection (real-time and resource-efficient).	2024
YOLOv8-HSFPN ^20^	Integrates High-level Screening Feature Pyramid Networks (HS-FPN), dynamically fusing features across resolutions to balance small and large object detection.	Combines shallow and deep feature extraction with channel attention mechanisms to address visual discrepancies within the same class.	Medical imaging	2024
YOLOv8-goldyolo ^25^	Implements the Gather-and-Distribute (GD) mechanism, redistributing multi-scale features via convolution and self-attention for enhanced resolution handling.	Applies MAE-style pre-training on ImageNet to strengthen feature representation and reduce sensitivity to intra-class variance.	General-purpose object detection (edge devices, robotics).	2023
YOLOv8-CFPT-P2345 ^23^	Leverages the Cross-Layer Feature Pyramid Transformer (CFPT) to apply spatial and channel-wise attention for precise multi-scale feature refinement.	Uses Cross-Layer Channel-wise Attention (CCA) and Cross-Layer Spatial-wise Attention (CSA) to highlight distinguishing features within classes.	UAV-based aerial images.	2024
YOLOv8-bifpn ^21^	Integrates Bi-Directional Feature Pyramid Network (BiFPN) to enhance feature fusion across scales with learnable weight assignments for accuracy.	Flexible fusion of multi-scale features ensures distinct class-based feature preservation during detection.	General-purpose object detection (efficient multi-resolution).	2023
YOLOv8-ASF-P2 ^22^	Employs Attentional Scale Sequence Fusion (ASF) to focus on scale-relevant features, enabling efficient sequence-based multi-scale detection.	Uses Channel and Position Attention Mechanisms (CPAM) to emphasize discriminative features for small object differentiation.	Medical imaging (e.g., cell segmentation).	2023
YOLOv10 ^24^	Adopts dynamic scaling mechanisms and optimized block design for multi-scale adaptability in detection.	Introduces consistent dual assignments for NMS-free training, enhancing feature alignment and differentiation.	General-purpose object detection (real-time efficiency).	2024
APF-YOLO	Combines Adaptive Path Fusion (APF) with P2-P5 layer connections and the Adaptive Feature Alignment Module (AFAM) for comprehensive multi-scale integration.	Deep semantic feature extraction via large path in AFAM.	UAV-based inspections (power lines and insulator detection).	2024

## Methods

### A. Problem statement

Modern YOLO models adopt a three-component architecture comprising a backbone, neck, and head. While the backbone extracts spatial and semantic features and the head generates predictions, the neck is pivotal in bridging these two components. By fusing multiscale features, the neck ensures the detection of objects of varying sizes and appearances, making it a critical area for improvement in complex detection tasks.

In the context of UAV-based power line inspections, detecting insulators presents unique challenges due to significant intra-class variance. Insulators—composed of materials like glass, composite, and porcelain—share a similar global shape but differ subtly in material texture and appearance. This poses a dual challenge: the model must preserve high-level semantic information to recognize shared global shapes while capturing fine-grained spatial details to differentiate between types.

The neck plays a vital role in this process by managing multiscale feature fusion. For insulators, preserving semantic consistency during feature fusion is essential to handle intra-class variance effectively. The global shape must be retained to ensure the model recognizes insulators as a single class, as insulators generally share a similar global shape regardless of their type. Standard architectures like PAN-FPN offer a partial solution to these challenges but leave room for optimization, as they often struggle to achieve a perfect balance between fine-grained details and high-level semantic features.

To resume, by focusing on neck improvements (not backbone or head), our approach seeks to robustly address multi-scale and intra-class variance challenges, ensuring accurate and reliable insulator detection in UAV-based power line inspections.

### B. Introduction to YOLOv8

YOLOv8 ^14^ represents a significant milestone in the evolution of the YOLO series, renowned for its exceptional performance in real-time object detection. Real-time detection refers to a model’s ability to swiftly and accurately identify and localize objects with minimal latency, a capability crucial for applications like autonomous driving, live video tracking, and UAV-based power line inspections YOLOv8’s balance of speed, accuracy, and efficiency makes it particularly well-suited for these time-sensitive tasks. ^26^

One of YOLOv8’s key strengths lies in its architecture, optimized for resource-constrained environments. It offers lightweight versions, such as nano and small-sized models, which achieve high performance even on devices with limited computational capacity. This adaptability is especially advantageous in UAV-based power line inspections, where computational efficiency and portability are essential for real-time anomaly detection.

YOLOv8 introduces several architectural innovations that enhance object detection performance. Its anchor-free head architecture decouples classification and localization, enabling the model to independently optimize these tasks. This decoupling improves both the accuracy of object identification and the precision of bounding box predictions. Advanced loss functions, such as CIoU Loss for bounding box regression, ^27^ DFL (Distribution Focal Loss) ^28^ for improved classification, and VFL (Varifocal Loss) ^29^ for addressing class imbalance, further refine the model’s detection capabilities.

The introduction of the C2f module marks another major improvement over its predecessor, the CSP (Cross-Stage Partial) layer in YOLOv5. By enabling more efficient feature integration and better context understanding, the C2f module enhances YOLOv8’s ability to manage multiscale detection. This is particularly relevant for detecting objects like insulators, which vary in size and appearance but share similar global shapes, as in UAV inspection tasks.

Another notable advantage of YOLOv8 is its active development within the open-source community. ^30^ Regular updates and contributions ensure that the model evolves alongside advancements in object detection, maintaining its position at the forefront of the field. This ongoing support also facilitates its adaptability to various applications, from industrial monitoring to UAV operations.

In conclusion, YOLOv8 serves as an excellent baseline model for real-time object detection tasks, offering a robust combination of speed, accuracy, and efficiency. These strengths underscore YOLOv8’s suitability for our study, where robust detection performance is crucial in managing multiscale objects and subtle material differences in UAV-based power line inspections.

### C. The architecture of YOLOv8

The YOLOv8 architecture integrates a Path Aggregation Network combined with a Feature Pyramid Network (PAN-FPN) as its neck component, crucial for addressing multiscale object detection challenges. The PAN-FPN establishes a connection between the backbone, responsible for extracting feature maps, and the detection head, which generates the final predictions. Its primary function is to fuse multiscale features, ensuring that the model can accurately detect objects of various sizes by combining both high-resolution and low-resolution information.

**Figure 2.**
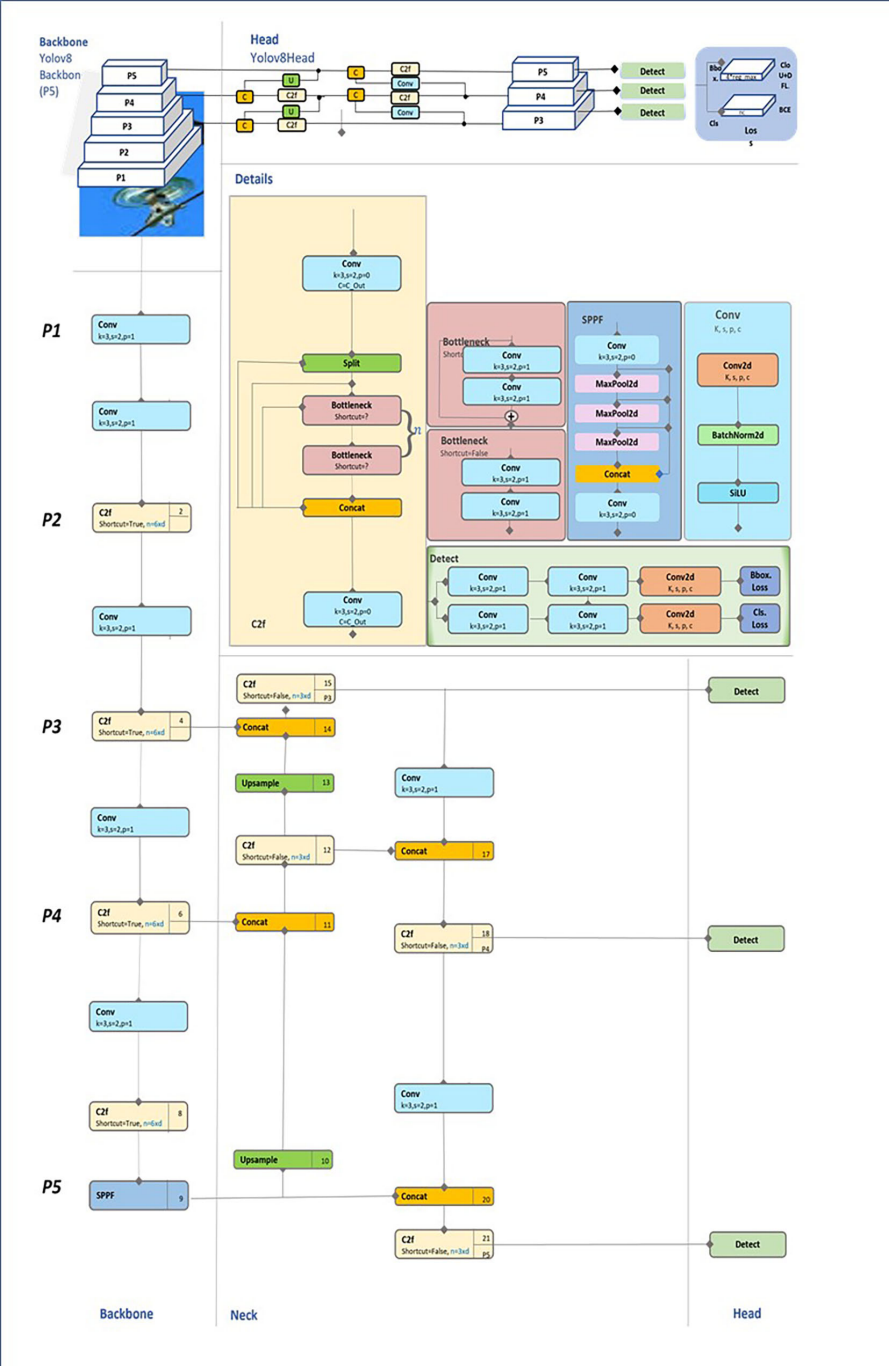
Diagrammatic representation of YOLOv8 network architecture.

This section will provide an explanation of the PAN-FPN neck, supported by Figure 2, which illustrates the feature propagation from the backbone to the detection head. We begin with an overview of the backbone and detection head, followed by a detailed discussion of the neck, which serves as the primary focus of this section due to its pivotal role in improving multiscale/intra class variance object detection.

Backbone Overview

The backbone in YOLOv8 is responsible for extracting hierarchical features from the input image at various levels of abstraction, producing feature maps at multiple resolutions. Lower layers of the backbone focus on spatially detailed information (high resolution), whereas deeper layers capture semantic-rich, low-resolution features. However, these feature maps need to be combined effectively for robust object detection across multiple scales. This is where the PAN-FPN neck plays a critical role, merging these multiscale features to ensure that both small and large objects are detected with high precision. In the context of Figure 2, the backbone is represented on the left, with feature maps being processed at various stages (P3, P4, P5) before entering the neck component.

The Role of PAN-FPN in the Neck

The PAN-FPN neck introduces a pyramidal structure that facilitates both bottom-up and top-down feature propagation, as seen in the central portion of Figure 2. This bidirectional flow is designed to capture fine-grained spatial details while maintaining high-level semantic context, which is essential for detecting objects of different sizes in complex scenes.

*Bottom-Up Pathway (From P5 to P3)*

The bottom-up pathway begins with P5, the deepest feature map output by the backbone, characterized by low spatial resolution but rich semantic information. This pathway progressively refines spatial detail and propagates semantic richness upward:

Upsampling: The Upsample Block (e.g., Block 10 in Figure 2) performs a 2x upsampling of the P5 feature map to match the resolution of the P4 map. This operation enlarges the spatial dimensions while preserving semantic information.

Concatenation: The upsampled P5 features are concatenated with the P4 feature map via a Concat Block (Block 11). This fusion integrates high-resolution spatial information from P4 with the semantic depth of P5, enhancing the network’s ability to handle multiscale objects.

C2f Block Refinement: After concatenation, the combined features pass through a C2f Block (Block 12). This block applies lightweight convolutions and shortcut connections to refine the fused feature map, balancing computational efficiency with feature enhancement.

SPP Module: At the P5 stage, the SPP (Spatial Pyramid Pooling) Module (Block 9) pools features at multiple scales to expand the receptive field, enabling the network to detect large objects requiring broader contextual understanding.

*Top of the Pyramid (P3)*

At the top of the pyramid, the network processes P3, the highest-resolution feature map. This stage prioritizes fine-grained spatial detail for detecting smaller objects:

Upsampled Features: The upsampled P4 features (processed through Block 10) are fused with the high-resolution P3 features via a Concat Block (Block 17). This operation ensures that both small-scale spatial details and intermediate semantic information are preserved.

Semantic Enrichment: The fused features pass through another C2f Block (Block 18) to enhance feature representation while maintaining a lightweight computational footprint. This integration ensures that small objects benefit from both spatial precision and the semantic depth retained from deeper layers.

*Top-Down Pathway (From P3 to P5)*

The top-down pathway propagates high-level semantic information downward, ensuring that all layers contribute to detecting objects of various sizes. This involves: Downsampling through Convolution: The high-resolution P3 feature map is downsampled via a Conv Block (e.g., Block 14) with a stride of 2. This operation reduces spatial resolution while enhancing semantic richness, preparing the features for fusion with deeper layers (e.g., P4 and P5).

Feature Fusion: The downsampled P3 features are concatenated with the P4 features using a Concat Block (Block 20). Similarly, the P4 features are processed and concatenated with the P5 features through Concat Block (Block 21).

C2f Refinement: After each concatenation, the fused feature maps pass through C2f Blocks (e.g., Blocks 18 and 21). These blocks apply convolutional refinements and shortcuts to maintain spatial and semantic balance across scales.

To resume the neck, The PAN-FPN’s bidirectional flow is really important in handling multiscale objects thanks to a Bottom-Up Pathway (That captures spatially detailed information from deeper layers (P5 to P3) through upsampling and concatenation, ensuring smaller objects are accurately detected) and a Top-Down Pathway (That reintroduces high-level semantic information (P3 to P5) through downsampling and concatenation, improving contextual understanding for larger objects.)

Detection Head Overview

The detection head (depicted in the far-right section of Figure 2) is responsible for generating the final predictions, including object classes, bounding boxes, and confidence scores. The detection head leverages the fused feature maps produced by the PAN-FPN to ensure accurate predictions across different object scales. By utilizing multiscale feature maps, the detection head can identify both small and large objects with improved precision. Figure 2 illustrates how the multiscale information flows into the detection head, highlighting the importance of fused feature representations in producing accurate predictions.

In conclusion, the PAN-FPN neck in YOLOv8 plays a pivotal role in enabling efficient multiscale feature fusion, handling intra-class variance, and improving the overall detection capability of the model. Through its bottom-up and top-down pathways the PAN-FPN achieves a balanced integration of spatial and semantic information, which is critical for detecting objects of various sizes. The bidirectional feature fusion mechanism ensures that the network remains robust in complex detection scenarios, making PAN-FPN an indispensable part of YOLOv8’s performance in diverse environments.

### D. Our model-Adaptive Path Fusion YOLO (APF-YOLO)

The YOLOv8 model has achieved remarkable advancements in object detection, offering significant improvements in speed and accuracy. However, it faces notable challenges in handling multiscale objects and intra-class variance, particularly under the complex environmental conditions of UAV-based power line inspections. This is primarily because YOLOv8 was not specifically designed to address the unique challenges of such applications. To overcome these limitations, we introduce APF-YOLO (Adaptive Path Fusion YOLO), which focuses on tackling the dual challenges of multiscale object detection and intra-class variance. By incorporating innovative architectural changes and advanced feature fusion strategies, APF-YOLO enhances the detection of both small and large objects while effectively differentiating visually similar objects within the same class. Below, we outline the core methodology and contributions of APF-YOLO:

Multiscale Object Detection

Detecting objects of varying sizes in UAV imagery presents significant challenges due to the wide range of spatial scales. Small objects require precise attention to fine-grained details, while larger objects demand an understanding of broader contextual relationships. To address these challenges, APF-YOLO introduces the Adaptive Feature Alignment Module (AFAM), a key innovation designed to dynamically align and fuse feature maps from three consecutive layers (e.g., P3, P4, P5).

**Figure 3.**
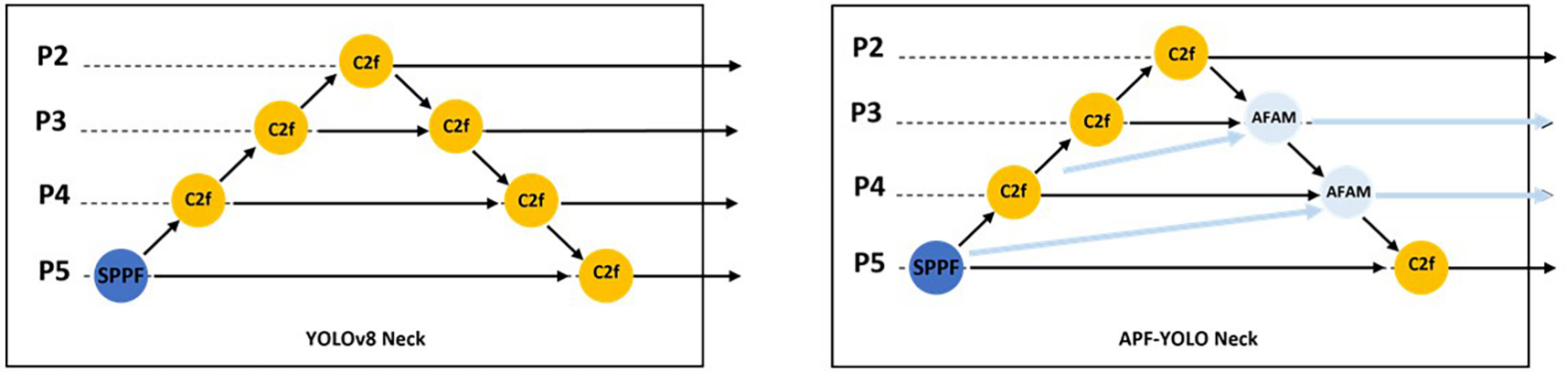
Schematic comparison of YOLOv8 Neck and APF-YOLO Neck.

**Figure 4.**
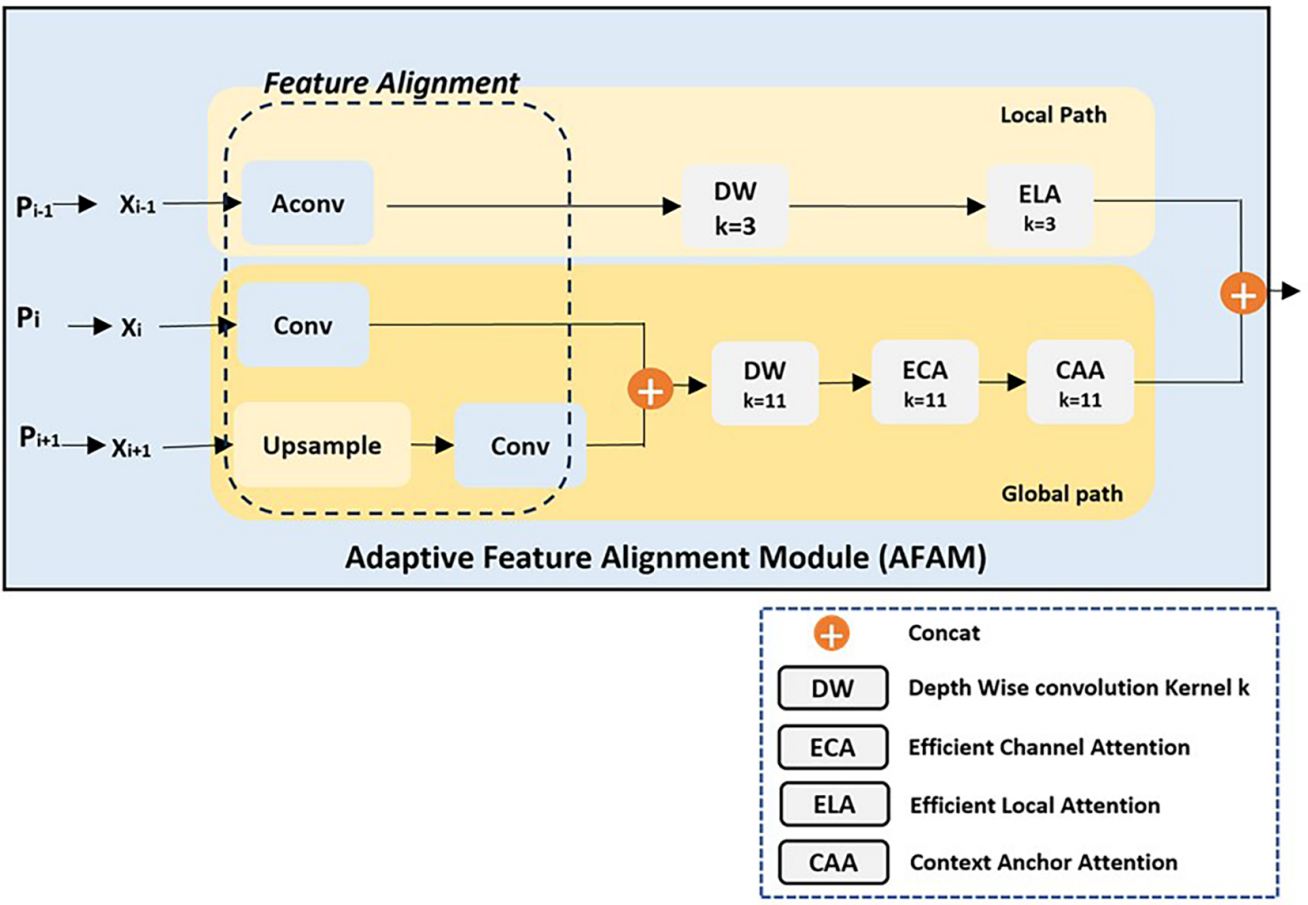
Architecture of the Adaptive Feature Alignment Module (AFAM) with Local and Global Pathway.

As shown in Figures 3 and 4, AFAM uses a flexible Feature Alignment process that adjusts the three input feature resolutions to a common scale, with the mid-layer (e.g., P4) serving as the reference resolution. This dynamic adjustment ensures that multiscale features are effectively combined, regardless of the specific input layers. The intuition behind Feature Alignment stems from the observation that feature maps from different layers inherently differ in resolution and semantic content. High-resolution maps (e.g., P3) capture fine spatial details, while low-resolution maps (e.g., P5) provide broader semantic context. Combining these directly without alignment can distort the fused representation, as the differing spatial scales introduce inconsistencies. To address this, AConv, an effective downsampling method from YOLOv9 authors, is used to reduce the resolution of higher-resolution inputs (e.g., P3) while preserving critical spatial features. Simultaneously, upsampling techniques are applied to low-resolution maps (e.g., P5) to bring them to the mid-layer’s resolution. This alignment step not only ensures consistency but also enables seamless fusion of features across scales.

Once aligned, the feature maps are processed through two complementary pathways: the local pathway and the global pathway. The local pathway focuses on fine-grained details from the highest-resolution input (e.g., P3). It applies Depth-Wise Convolutions (DW, k=3) to capture localized patterns efficiently and Efficient Local Attention (ELA, k=3) to refine these features by selectively enhancing critical spatial details. The intuition behind the local pathway is that small objects, such as insulators captured from a distant perspective, require precise spatial detail preservation, which this pathway is explicitly designed to provide. In contrast, the global pathway processes the semantically rich but lower-resolution inputs (e.g., P4 and P5) to capture broader contextual relationships. This pathway starts with Depth-Wise Convolutions (DW, k=11) to expand the receptive field, capturing structural and spatial information across the scene. The global pathway then applies Efficient Channel Attention (ECA) to emphasize the most semantically meaningful feature channels, ensuring that high-level structural attributes are prioritized. Finally, Context Anchor Attention (CAA) refines these features by focusing on spatial and contextual relationships, enabling the model to understand how different parts of the object and its surroundings interact. The intuition behind this pathway is that larger objects and their contexts require broad, high-level understanding that complements the localized detail captured in the local pathway.

The outputs from both pathways are fused through concatenation, creating a unified feature representation that balances spatial precision and semantic richness. This fusion ensures that small objects are detected with high-resolution detail while larger objects and their contextual relationships are captured through broader semantic understanding.

By dynamically aligning feature maps and combining complementary pathways, AFAM is designed to handle multiscale detection challenges effectively, making it particularly suitable for UAV-based applications like power line inspections, where detecting objects of varying sizes and understanding their spatial context are paramount.

To further improve small object detection, we introduced a direct pathway from P2 (backbone) to the neck (Refer to Figure 3). By incorporating this high-resolution feature map, the model gains access to additional fine-grained information, enabling it to better identify and differentiate small objects in complex UAV imagery.

Intra-Class Variance Handling

Intra-class variance, where objects within the same class differ subtly in material or texture (e.g., glass, porcelain, and composite insulators), presents a significant challenge in object detection. These variations in material properties often arise from manufacturing differences or environmental exposure, yet the insulators retain a consistent global shape across classes. This shared structural feature serves as a key focus for addressing intra-class variance.

The Adaptive Feature Alignment Module (AFAM), specifically its global pathway, is designed to leverage this insight by emphasizing high-level semantic information extracted from deeper network layers (e.g., Pi and Pi+1). These layers inherently capture critical structural features such as shape and form, which are essential for distinguishing insulators that share similar appearances but vary in finer material-specific details. To preserve and refine this information, the global pathway begins with a Depth-Wise Convolution (DW, k=11), which expands the receptive field to capture broader spatial relationships. This operation ensures that global features representing the object’s overall shape and structural integrity are prioritized in the feature representation. The intuition behind this design is rooted in the idea that global shape consistency provides a robust framework for distinguishing objects within the same class, even when their textures or fine details differ. The global pathway complements this approach by incorporating advanced attention mechanisms, specifically Efficient Channel Attention (ECA) and Context Anchor Attention (CAA). These mechanisms are designed to address the subtle variations within a class that can manifest in specific feature channels or spatial regions. ECA selectively emphasizes the most relevant feature channels, ensuring that the representation focuses on high-level semantic characteristics like shape and structure while filtering out irrelevant or noisy details. This helps strengthen the representation of shared attributes across the class, reducing the risk of misclassification caused by misleading material-specific features. Meanwhile, CAA operates by capturing meaningful spatial and contextual relationships within the feature maps, ensuring that the global pathway effectively understands how different parts of the object relate to each other and their surroundings.

This spatial refinement is particularly valuable for objects like insulators, which often appear in complex or cluttered environments where contextual information is critical. The outputs of the global pathway, refined through DW, ECA, and CAA, focus on the global shape and semantic consistency of the objects. However, addressing intra-class variance with multi scale objects also requires integrating material-specific granularity. For this purpose, the refined outputs from the global pathway are fused with those from the local pathway, which captures fine-grained material details using high-resolution inputs (e.g., P2 or P3, depending on the scenario). The local pathway complements the global pathway by focusing on features that highlight differences in texture or material properties.

By leveraging these complementary pathways, APF-YOLO is designed to address the challenges of intra-class variance in UAV-based power line inspections, where insulators of different materials must be reliably detected. Future evaluations will assess how effectively this design meets its intended goals in real-world applications, focusing on its ability to balance global consistency and localized detail extraction.

More technical details regarding these components and their implementation can be found in the subsequent sections of this paper.

1) **Depth-Wise Convolution (DW)**

**Figure 5.**
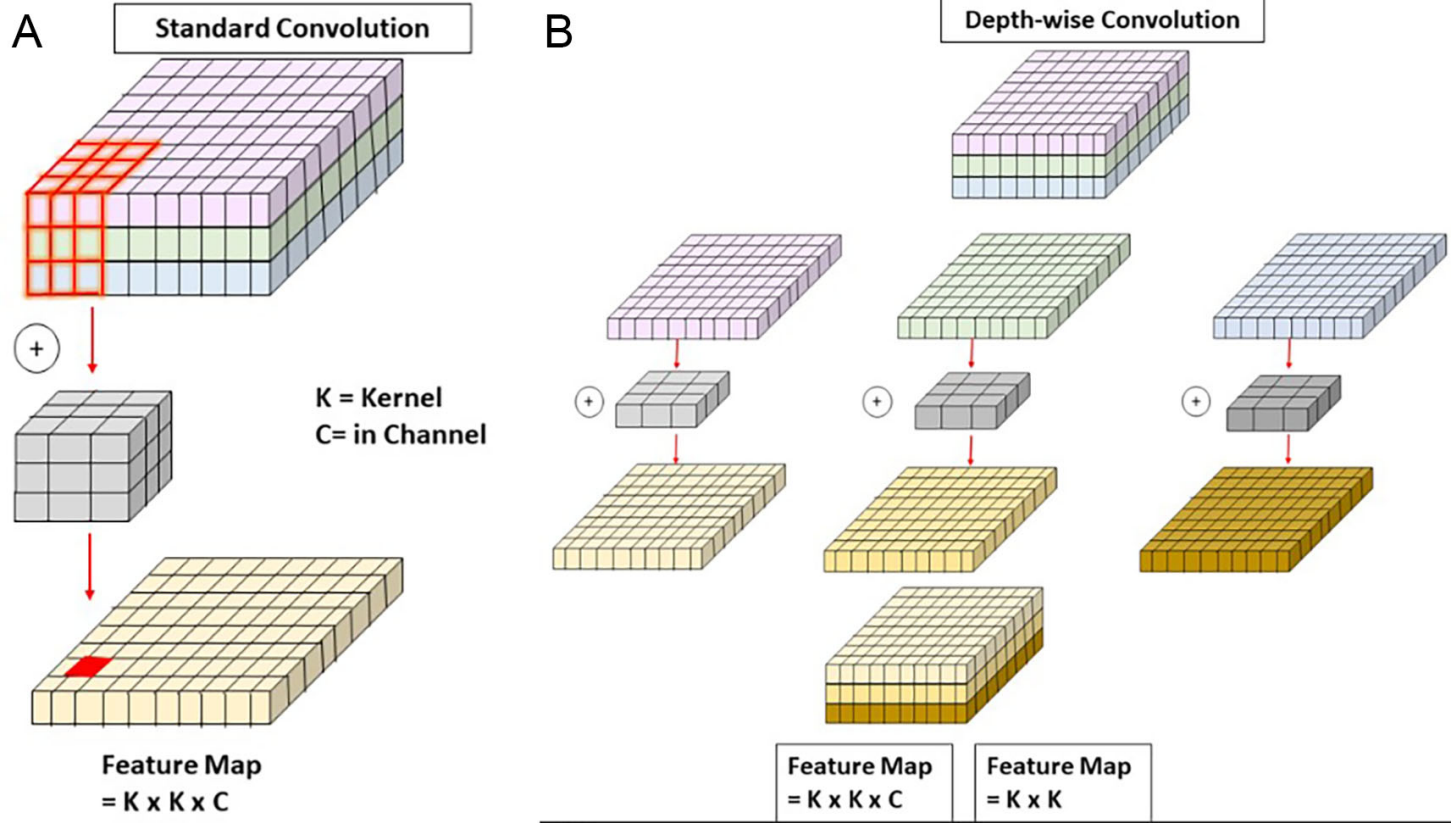
Schematic comparison of Standard Convolution and Depth-Wise Convolution.

Depth-Wise Convolution (DW), ^31^ as illustrated in Figure 5, offers a computationally efficient alternative to traditional convolution by processing each channel independently with separate filters. Unlike standard convolution, where a single kernel operates across all channels simultaneously (left side of the figure), DW applies a distinct K×K kernel to each channel individually and stacks the outputs (right side of the figure). This significantly reduces computational cost while retaining channel-specific features. For an input feature map of size H×W×C, the computational cost of DW is K ^2^ ×H×W×C, compared to K ^2^×H×W×C×N for standard convolution, where N is the number of output channels. In APF-YOLO, DW is applied with kernel sizes k=3 and *k* = 11, tailored for the distinct roles of the local and global pathways within the AFAM. The local pathway, designed to process high-resolution feature maps, uses a k=3 kernel. As shown in the figure, the smaller kernel size focuses on localized patterns while maintaining computational efficiency. This is critical for detecting small objects where fine-grained spatial detail is essential. On the other hand, the global pathway processes low-resolution feature maps using a k=11 kernel to expand the receptive field and capture broader spatial relationships. As depicted in the figure, the larger kernel size enables the model to aggregate information over a wider area, which is crucial for understanding the structure and context of larger objects. This is particularly important for addressing intra-class variance in insulators (e.g., glass, porcelain, and composite) by focusing on the global shape and form. Figure 5 illustrates how DW separates operations across channels (right) compared to the combined processing of all channels in standard convolution (left). The use of k=3 in the local pathway ensures high-resolution features remain precise, while k=11 in the global pathway emphasizes semantic context. This dual-kernel approach reflects the intuition behind APF-YOLO’s design. Together, they balance localized precision and contextual understanding, enabling the model to handle multiscale object detection and intra-class variance effectively.

2) **AConv**

**Figure 6.**
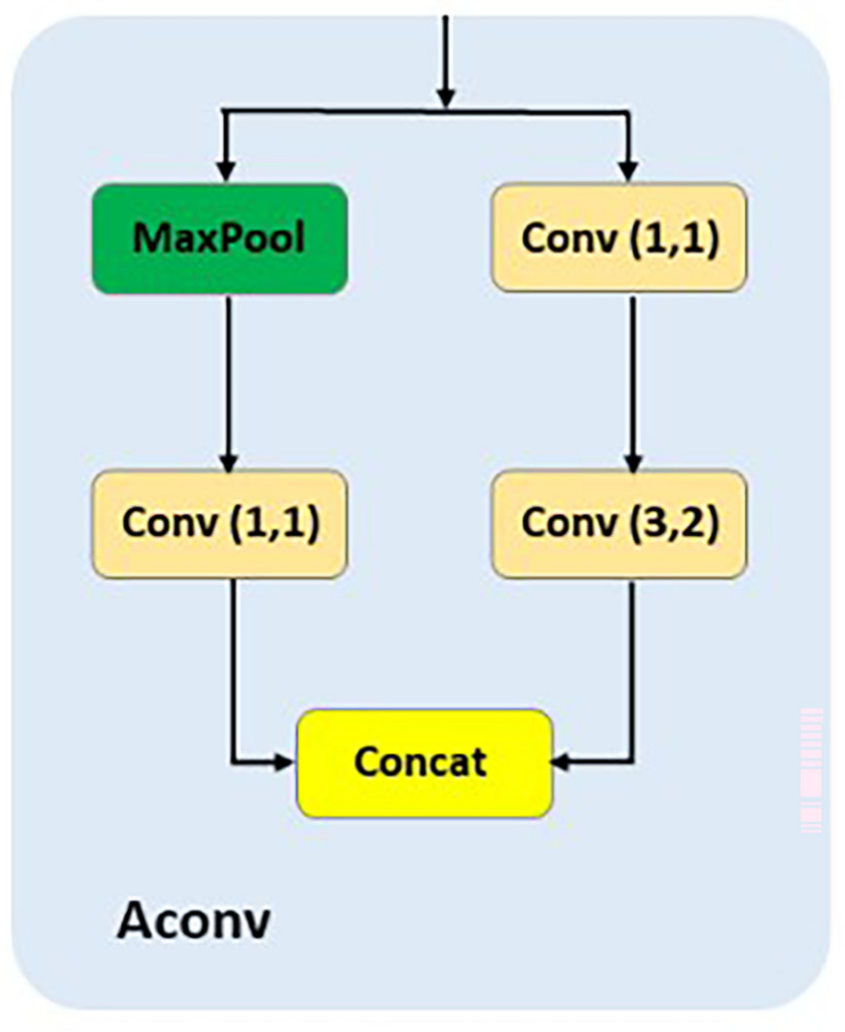
Schematic representation of Aconv.

The AConv module ^18^ is designed specifically for downsampling to facilitate feature alignment in multiscale architectures like APF-YOLO. As shown in the Figure 6, AConv combines max pooling, which reduces the spatial resolution of the input while preserving dominant features, and a 3×2 convolution with stride 2, which processes spatial information during downsampling. Additionally, two 1×1 convolutions are used to refine and adjust channel dimensions before the outputs are concatenated. The purpose of AConv in APF-YOLO is to resize the feature maps from the P(i−1) layer to match the dimensions of the mid-layer P(i), enabling seamless integration in the Feature Alignment stage.

3) **ELA**

**Figure 7.**
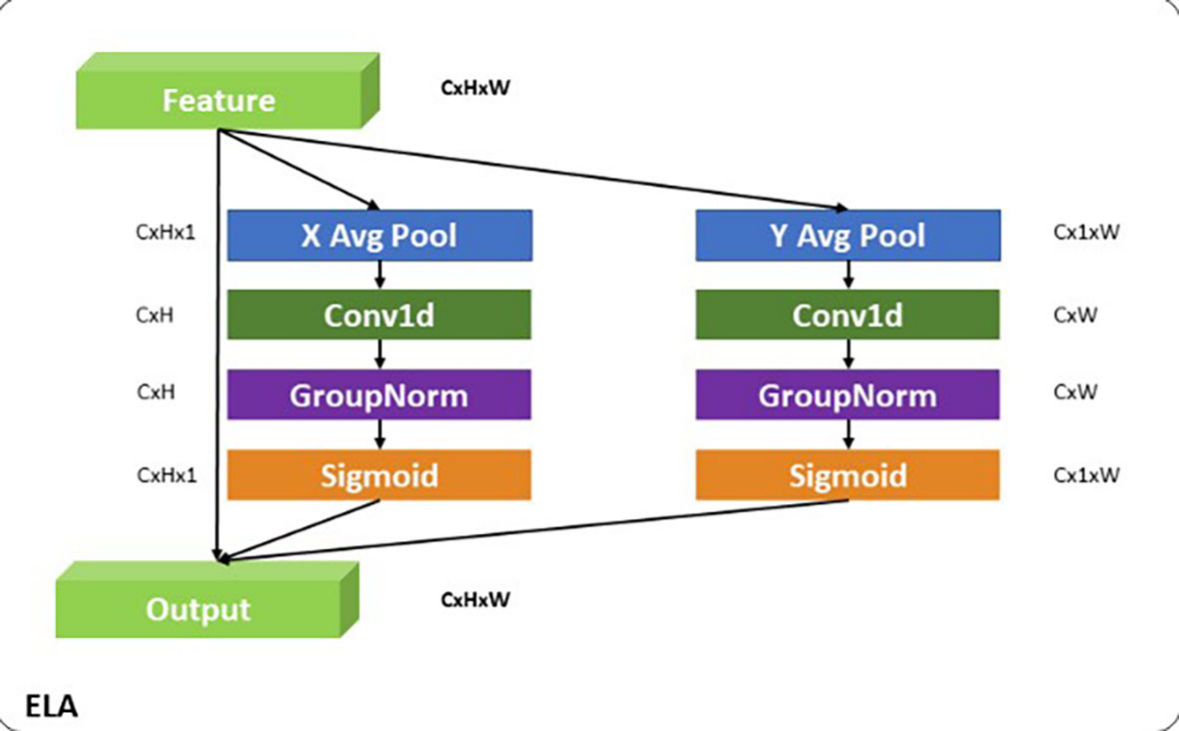
Diagrammatic representation of Efficient Local Attention (ELA).

The Efficient Local Attention (ELA) module ^32^ is designed to refine and selectively emphasize fine-grained spatial features critical for accurately localizing small objects like insulators. It is strategically placed after the Depth-Wise Convolution (DW, k=3) in the local pathway to build upon the initial spatial features extracted by the convolution. The DW convolution, with its (3×3) kernel, serves as the first stage, capturing localized spatial patterns and contours in a computationally efficient manner. This operation is particularly effective in extracting detailed features, such as the precise edges and shapes that are vital for localizing small-scale objects. However, DW operates uniformly across all spatial regions and channels, which may result in less focus on the most relevant localized features. To address this, ELA follows DW to enhance the extracted features by incorporating a selective attention mechanism. As shown in Figure 7, ELA begins with X and Y average pooling operations, which aggregate spatial information along the width and height dimensions, respectively, creating condensed intermediate representations that capture essential local spatial dependencies. These representations are then processed through (1×1) convolutions, enabling channel-wise interactions that enrich the feature representation while preserving critical spatial details. Group Normalization stabilizes the output, and a sigmoid activation function generates attention weights that are applied to the original feature maps. This process ensures that the most relevant spatial features are amplified while irrelevant or redundant information is suppressed. The intuition behind this design lies in the complementary roles of DW and ELA. While DW captures fine-grained spatial patterns, ELA refines these patterns by selectively focusing on the most important localized features. This sequential approach enables the local pathway in the Adaptive Feature Alignment Module (AFAM) to effectively combine spatial precision from DW with the selective focus of ELA. By doing so, APF-YOLO is designed to address the challenges of small object localization, ensuring that fine-grained spatial details are accurately captured and emphasized. This makes it particularly well-suited for UAV-based power line inspections, where precise localization of insulators across varying scales is a critical challenge.

4) **ECA**

**Figure 8.**
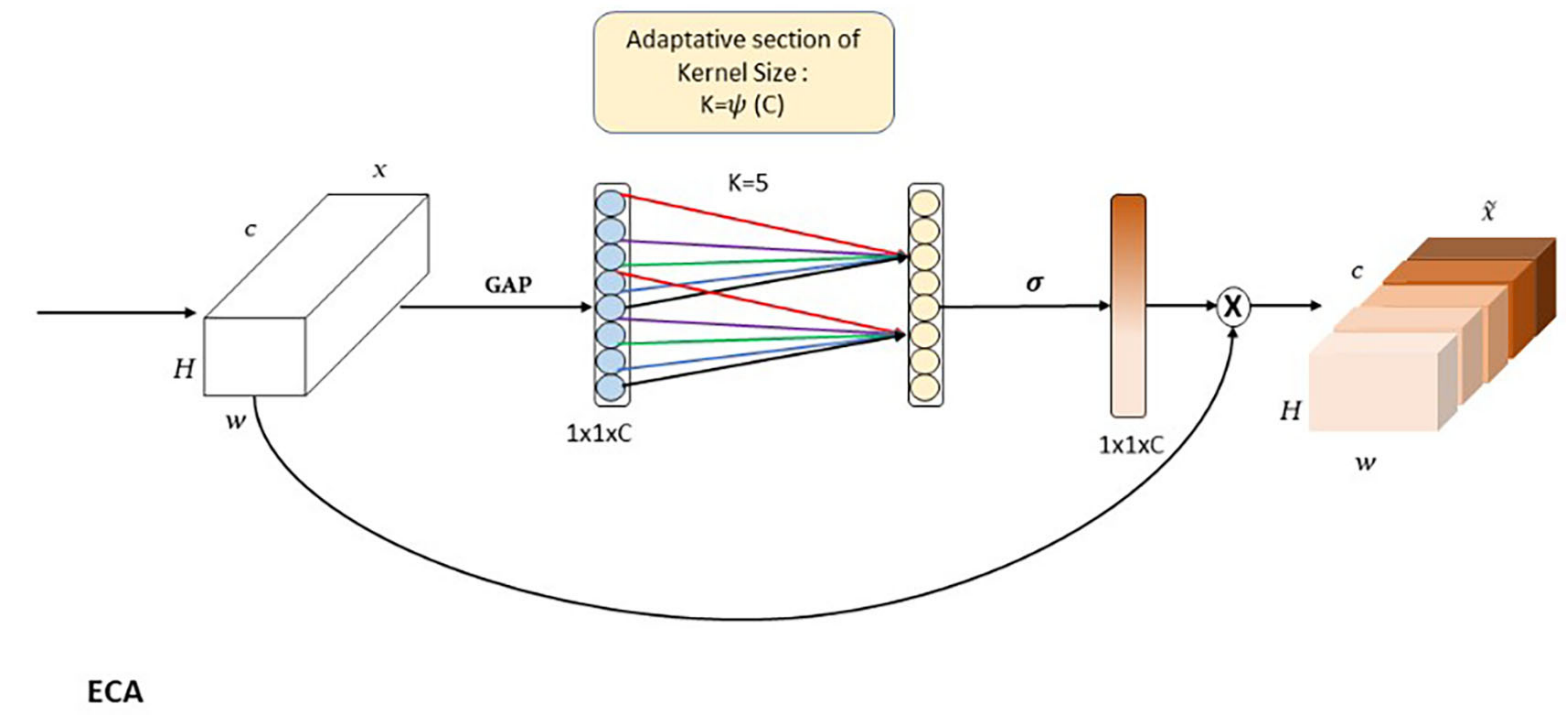
Diagrammatic representation of Efficient Channel Attention (ECA).

The Efficient Channel Attention (ECA) module ^33^ is designed to optimize attention across feature map channels, enabling the model to focus on the most informative features while suppressing less relevant ones. As shown in the Figure 8, ECA dynamically learns the importance of each channel and applies adaptive weighting to enhance feature representation. This capability is particularly critical for capturing high-level semantic features, essential for handling intra-class variations, such as differentiating between glass, porcelain, and composite insulators. ECA begins with Global Average Pooling (GAP), which aggregates spatial information (H×W) across each channel of the input feature map. This operation produces a condensed representation of size (C×1) , where (C) is the number of channels, summarizing the contribution of each channel. Following this, ECA applies a (1×1) convolution to learn inter-channel dependencies, with an adaptive kernel size determined k=ψ(C) , where (ψ(C)) is a mapping function. This adaptive mechanism is especially critical in lower layers, such as P5, which contain a large number of feature maps (i.e., channels). In these layers, the abundance of channels increases the likelihood of redundant or irrelevant information. Channel attention provided by ECA ensures that the model focuses on the most relevant features, filtering out less useful information. After the convolution, the output is passed through a sigmoid activation function, generating channel-wise weights that scale the original feature maps. This recalibration is expressed as (Y=X⊗s), where X represents the input feature map, s is the learned channel weight, and (⊗) denotes element-wise multiplication. By assigning greater focus to channels carrying significant semantic information and reducing the influence of irrelevant or noisy channels, ECA enhances the overall representation of feature maps. The ECA module follows the Depth-Wise Convolution (DW, k=11) in the global pathway for a specific reason. The DW convolution, with its large kernel size, captures broad spatial relationships critical for preserving the global shape and structure of objects. However, DW processes each channel independently and does not address inter-channel interactions. In deeper layers like (P5), which contain a high number of feature maps, this lack of channel interaction can dilute the representation’s efficiency. ECA complements DW by recalibrating the importance of each channel, ensuring that only the most relevant features are emphasized while redundant channels are suppressed. This sequence of operations enhances the global pathway’s ability to handle intra-class variance by focusing on structural and contextual features that are semantically meaningful.

In APF-YOLO ’s Adaptive Feature Alignment Module (AFAM), ECA plays a crucial role in refining channel-level representations, especially in layers with a high channel count. By leveraging ECA after DW, the global pathway is designed to balance spatial relationships captured by DW with the selective focus of channel attention, ensuring that the model can emphasize critical semantic features while reducing noise. This design ensures that APF-YOLO is meant to effectively handle complex scenarios, such as distinguishing between objects with subtle material differences, while preserving global structural integrity.

5) **CAA**

**Figure 9.**
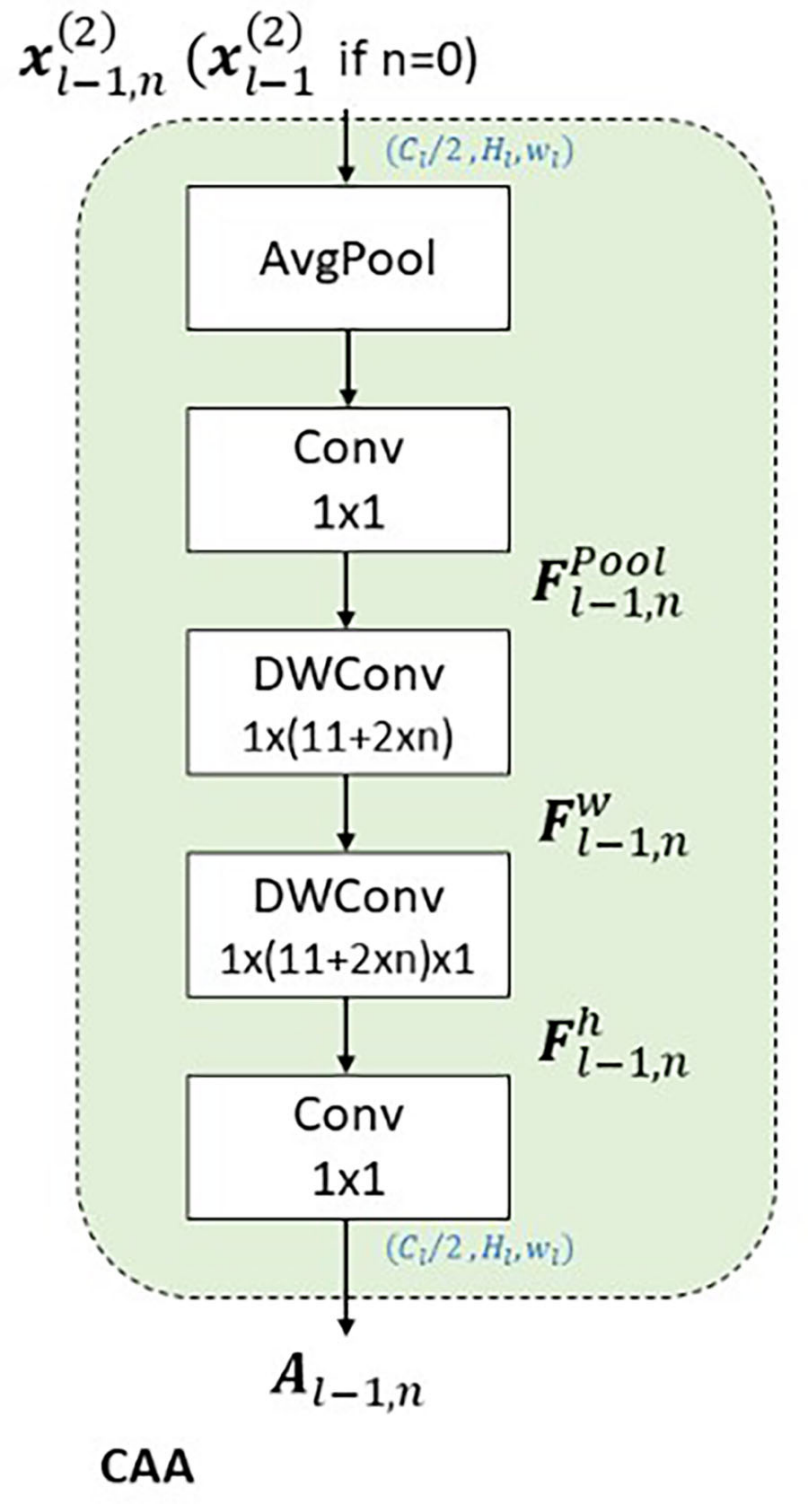
Diagrammatic representation of Context Anchor Attention (CAA).

**Figure 10.**
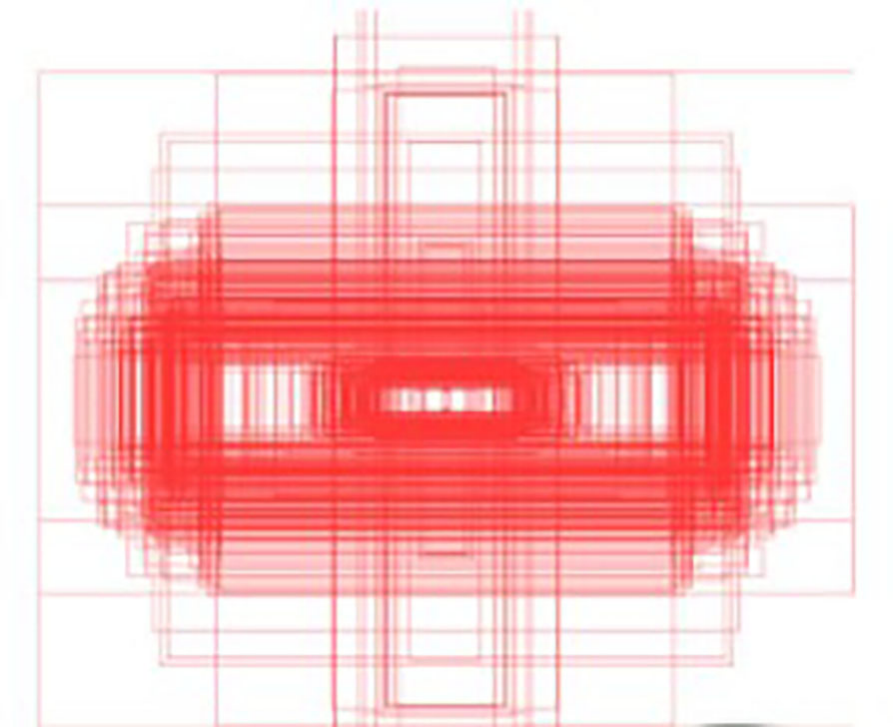
Visualization of Dataset Bounding Box Labels.

The Context Anchor Attention (CAA) module ^34^ introduces a context-aware mechanism designed to enhance the model’s ability to understand spatial relationships and interactions within a scene. This capability is particularly critical in scenarios involving insulators, which typically exhibit elongated rectangular shapes. As shown in the Figure 10, the bounding box annotations reveal that insulators predominantly align along horizontal and vertical axes. This characteristic directly informs the design of CAA, which employs depth-wise convolutions with kernels explicitly tailored for vertical ((1×(11+2n))) and horizontal (((11+2n)×1))contexts . By expanding the receptive field in these directions, CAA enables the model to analyze spatial dependencies and interactions specific to insulators’ orientation and geometry. As shown in the Figure 9, The CAA module begins by applying Average Pooling to aggregate spatial information from the input feature maps, reducing dimensions while retaining critical channel-wise information. The pooled features are processed through a (1×1) convolution, generating context anchors that model inter-channel relationships and lay the foundation for contextual refinement.

Following this, the vertical and horizontal depth-wise convolutions are applied to capture directional spatial relationships. These operations ensure that the model can effectively learn patterns corresponding to the insulator’s shape and context, such as alignment with power lines or placement within complex backgrounds. The outputs from the convolutions are refined through a final (1×1) convolution and normalized with a sigmoid activation function, producing attention weights that prioritize the most contextually relevant features. These weights enhance the model’s focus on spatial relationships critical for distinguishing insulators even when partially obscured or fragmented. The ability to analyze the insulator’s context along its natural horizontal and vertical axes ensures that spatial integrity is maintained, making CAA particularly effective in scenarios where accurate localization and classification depend on understanding the object’s surroundings. CAA complements the preceding Efficient Channel Attention (ECA) module in the global pathway. While ECA recalibrates channel relevance to prioritize semantically meaningful features, it does not address spatial dependencies. CAA builds on ECA by introducing spatial context awareness, refining the model’s ability to understand an insulator’s position and alignment within its scene. Together, these modules create a sequential process where features are first optimized for channel relevance and then enriched with spatial and contextual refinements. As depicted in the Figure 10, the rectangular shape and alignment of insulators strongly support the rationale for CAA’s design. Its focus on vertical and horizontal spatial relationships ensures that the model captures critical contextual information necessary for accurate detection, even when insulators are partially occluded or split due to background interference. This approach makes CAA well-suited for UAV-based power line inspections, where understanding both object structure and its spatial context is essential for precise localization and classification. In summary, APF-YOLO is built directly on the YOLOv8 architecture, with modifications made solely to the neck component to enhance multiscale feature fusion and address intra-class variance. The backbone, detection head, loss functions, and other components remain unchanged, leveraging YOLOv8’s inherent strengths while focusing improvements where they are most needed.

### E. Dataset description

**Figure 11.**
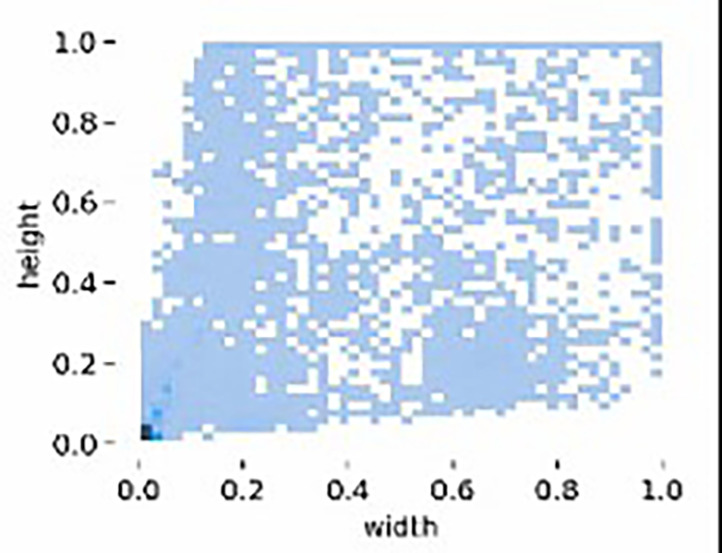
Distribution of Object Bounding Box Aspect Ratios (Height vs. Width).

**Figure 12.**
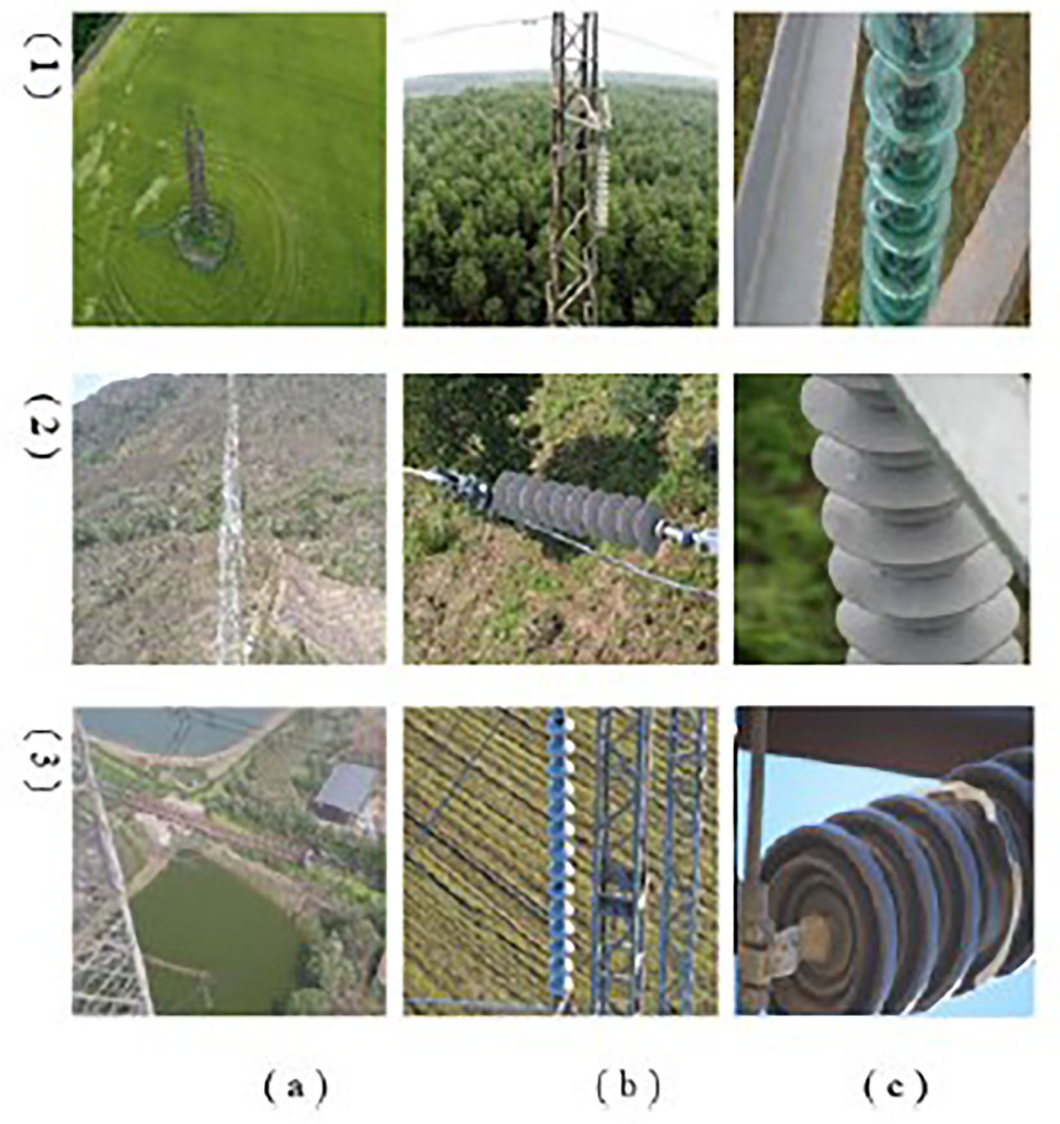
Sample images of the MPID. We propose 3 types of insulators, (1) glass (2) composite and (3) porcelain. With different distances of shooting by the drone (a) distant (b) full view (c) close up.

For our study, The Merged Public Insulator Dataset (MPID) ^52^ was constructed to address the need for a diverse, high-quality dataset specifically tailored for UAV-based high-voltage power line inspections. This dataset reflects real-world variability and provides a robust foundation for developing advanced object detection models, particularly those addressing multiscale object detection and intra-class variance challenges. Comprising 4807 images and 7850 labeled instances of three insulator types—glass, porcelain, and composite—as detailed in Table 3, MPID integrates multiple publicly available datasets from different regions worldwide as detailed in Table 2. This ensures a rich diversity of environmental and inspection scenarios, making it a practical and generalizable resource for power line inspection research. MPID includes images captured under diverse conditions, such as varying times of day, weather environments, lighting conditions, and camera angles, replicating the challenges faced during UAV inspections. The dataset also provides images from different distances and perspectives: (a) distant views, (b) full views, and (c) close-ups, as illustrated in Figure 11. This variability in viewpoints ensures that MPID reflects real-world conditions, addressing the inherent challenges posed by insulator scale variability in UAV imagery. Furthermore, the width-height distribution of insulators, visualized in Figure 12, highlights the multiscale problem inherent in MPID. The range of bounding box sizes indicates a broad spectrum of object dimensions, ensuring that models trained on MPID must detect both small and large insulators effectively (The darker blue regions indicate a higher concentration of insulators with those particular width-height ratios). This is particularly relevant for APF-YOLO, which aims to address multiscale detection by preserving critical information. MPID emphasizes three distinct types of insulators—glass, porcelain, and composite—which share a consistent global shape but differ in material and texture, introducing significant intra-class variance. Glass insulators are characterized by their transparency, porcelain insulators exhibit a ceramic texture, and composite insulators feature polymeric materials. This diversity challenges detection models to preserve semantic information while distinguishing between these visually similar objects. The inclusion of this variety makes MPID particularly relevant for developing models capable of managing intra-class variability. The dataset construction involved a semi-automatic annotation process to ensure high-quality labels. After filtering irrelevant images, pre-trained object detection models were used to generate initial bounding boxes, which were then manually refined using the Roboflow platform. ^44^ Annotators ensured that bounding boxes tightly encapsulated each insulator and accurately identified its type. The annotations were formatted in the YOLOv8 annotation standard, enabling seamless compatibility with YOLO-based models. To standardize the dataset, all images were resized to 640 × 640 pixels, the default input size for YOLO architectures. Unlike traditional dataset preparation processes, no manual data augmentation was applied during MPID construction. Instead, YOLOv8’s built-in augmentation capabilities—such as random scaling, rotation, and color adjustments—were leveraged during the training pipeline. This ensures that the model benefits from diverse variations in data without requiring explicit augmentation during dataset preparation. The use of YOLOv8’s augmentation mechanisms reduces preprocessing efforts while enhancing generalization during training. Furthermore, a typical 80-20 train-validation split was applied to the dataset to facilitate model evaluation. By offering a diverse and realistic dataset, MPID supports the development of advanced detection models. Its focus on multiscale variability and intra-class diversity ensures that models trained on it are equipped to handle the complexities of real-world UAV-based power line inspections.

**Table 2.** List of public datasets used to create MPID.

Name by Authors	Source	Country	Number of Images	Type of Insulator	Presence of Anomaly	Resolution in pixel*pixel
CPLID	^35^	China	848	Porcelain	Yes	At least 1000*800
STNPLAD	^36^	Brazil	324	Composite	Yes	At least 5472*3648
IDID	^37^	United States	1600	Porcelain	Yes	At least 1600*1080
Pylon-components-dataset	^38^	Denmark	555	Glass	Yes	At least 3000*1800
Sut110x-polgyon	^39^	Vietnam	87	Glass/Composite	Yes	515*515
Dataset-Insulators-Neering	^40^	Unknown	104	Porcelain	Yes	608*608
Insulator-2	^41^	Unknown	135	Glass	Yes	1280*1280
Insulator-full	^42^	Unknown	394	Composite	Yes	512*512
Long-view	^43^	Unknown	163	Glass/Composite	Yes	At least 2200*1700

**Table 3.** Distribution of instances and images by insulator type in MPID.

Insulator type	Number of instances	Number of images
Glass	3773	1612
Porcelain	3172	2549
Composite	905	646
All	7850	4807

## Experiments

### A. Environment configuration

The training and validation processes for this study were conducted in a carefully designed computing environment tailored to the computational demands of real-time object detection tasks. The experiments utilized a virtual machine equipped with a Quadro P6000 GPU, offering 24 GB of VRAM. This GPU’s high memory capacity and parallel processing capabilities ensured efficient handling of the large multiscale dataset and computationally intensive training of deep learning models. CUDA version 12.5 was employed to leverage GPU acceleration, streamlining algebraic operations critical for optimizing neural network training.

The software environment was built on Windows 10, chosen for its stability and compatibility with diverse machine learning tools. Python 3.9.10 served as the programming language, enabling seamless integration with the PyTorch version 2.2.2+cu121 which was selected for its flexibility and robust support for neural network development, making it particularly suitable for adapting and fine-tuning YOLO models. Model development and training were further facilitated by Ultralytics’ tools, optimized for YOLOv8 models and designed to enhance both speed and accuracy in detection tasks.

To ensure efficient management and reproducibility of experiments, the MLOps platform ClearML ^45^ was employed. ClearML provided a centralized system for tracking experiments, monitoring metrics, and collaborating across development stages, supporting the study’s iterative experimentation process.

This computing environment not only ensured smooth training and validation but also provided a foundation for reproducibility, scalability, and efficiency throughout the experimental process.

### B. Models

This section outlines the models chosen for comparison, focusing specifically on state-of-the-art (SOTA) neck configurations within the YOLO family. These configurations are evaluated for their ability to address key challenges in object detection, such as multiscale detection and intra-class variance. By comparing a variety of neck designs, the study aims to understand their strengths and weaknesses in handling these complexities.

A diverse set of models is included in the analysis, each representing unique design philosophies for improving feature fusion and context understanding. These models include YOLOv8n_baseline (default YOLOv8 neck), YOLOv8n-p2 (incorporating the P2 layer for small object detection), YOLOv8n-MAFPN, YOLOv8n-HSFPN, YOLOv8n-goldyolo, YOLOv8n-CFPT-P2345, YOLOv8n-bifpn, and YOLOv8n-ASF-P2. For additional perspective, the study also considers YOLOv6n, YOLOv5n, YOLOv9-t, and YOLOv10n models, representing variations in architecture and size.

The models chosen are primarily in the “n” (nano) size category, which are lightweight configurations optimized for edge devices and consumer GPUs. This focus ensures that the analysis remains relevant to real-world applications, such as UAV-based power line inspections, where computational resources are often limited. Larger models, while offering higher precision due to increased parameter counts, are typically excluded as they do not align with the operational constraints of real-time UAV integration. The emphasis on nano models also reflects their suitability for deployment on devices with restricted hardware capacity, balancing efficiency and accuracy.

### C. Metrics

To provide a comprehensive assessment of model performance, it is generally considered appropriate to utilize several different evaluation metrics. For the evaluation of insulator defect detection models, we employ a suite of key metrics to ensure a thorough and nuanced understanding of performance. These metrics include Precision, Recall, Mean Average Precision (mAP) at different Intersection over Union (IoU) thresholds, inference time, and Giga Floating Point Operations per Second (GFLOPS). Each metric provides distinct insights into the model’s effectiveness and efficiency.

Precision P measures the accuracy of the model’s positive predictions and is defined as:

$$P=\frac{\mathrm{TP}+\mathrm{FP}}{\mathrm{TP}\;}$$ (1)

where TP represents the number of true positive detections and FP denotes the number of false positives. A true positive is counted when the IoU (Intersection over Union) between the predicted bounding box and the ground truth bounding box is greater than 0.5. IoU is a metric that quantifies the overlap between the predicted and actual bounding boxes. It is computed as:

$$\;\mathrm{IoU}=\frac{\text{Area of Union}}{\text{Area of Overlap}\;}$$ (2)

where the Area of Overlap is the area where the predicted and ground truth boxes intersect, and the Area of Union is the total area covered by both boxes combined. High precision indicates fewer false alarms, reflecting the model’s ability to correctly identify defect cases.

Recall R assesses the model’s capability to detect all relevant defect instances. It is computed as:

$$R=\frac{\mathrm{TP}+\mathrm{FN}}{\mathrm{TP}\;}$$ (3)

where FN represents the number of false negatives. High recall signifies the model’s effectiveness in identifying actual defect cases, minimizing missed detections.

Average Precision (AP) evaluates the model’s performance across different recall levels by integrating the precision-recall curve. The AP is computed as:

$$\mathrm{AP}=\int_{0}^{1}\;\mathrm{PdR}\;$$ (4)

Mean Average Precision (mAP) extends AP to multiple classes by averaging AP values across all categories. It is defined as:

$$\;\mathrm{mAP}=\frac{1}{k}\sum_{i=1}^{k}\mathrm{AP}i\;$$ (5)

where k is the number of defect categories and AP _i_ is the average precision for category i. mAP is particularly useful for evaluating model performance across diverse defect types (two in our case).

In addition to mAP, we assess the model using Average Precision (AP) at specific Intersection over Union (IoU) thresholds. We calculate AP at IoU = 0.5 (denoted as AP@0.5) and average AP across IoU thresholds from 0.5 to 0.95 in increments of 0.05 (denoted as AP@0.5:0.95). These metrics provide a detailed evaluation of the model’s performance with varying degrees of overlap between predicted and ground truth bounding boxes.

Inference time measures the duration required for the model to process an image and produce predictions. This metric is critical for real-time applications where rapid processing is essential. Inference time is expressed in milliseconds and reflects the model’s efficiency in practical scenarios.

GFLOPS quantifies the computational complexity of the model by indicating the number of floating-point operations performed per second. This metric helps assess the model’s computational demands and is crucial for understanding its performance capabilities. Higher GFLOPS values suggest more complex models, which may impact real-time processing.

These metrics collectively offer a thorough evaluation framework for assessing the performance, efficiency, and practicality of object defect detection models.

### D. Training process

Models are trained using standard parameters and hyperparameters recommended by the original authors, accessible via the Ultralytics platform. ^30^ This approach ensures consistency with established research standards, providing a fair and unbiased basis for comparison. To ensure unbiased performance evaluation, models are trained from scratch. For this study, we focus on various neck configurations, including YOLOv9-t, YOLOv8n_baseline, YOLOv8n-p2, YOLOv8n-MAFPN, YOLOv8n-HSFPN, YOLOv8n-goldyolo, YOLOv8n-CFPT-P2345, YOLOv8n-bifpn, YOLOv8n-ASF-P2, YOLOv6n, YOLOv5n, and YOLOv10n.

The training process involves 50 epochs with a batch size of 32, and the input image size is set to 640 pixels. Data augmentation techniques are automatically applied during training, enhancing the model’s robustness and enabling it to generalize better across varying conditions. The use of data augmentation is crucial, as it simulates a diverse set of scenarios, helping to improve the model’s ability to handle multiscale detection and intra-class variance. The total training duration for all models is approximately 11 hours, allowing for thorough evaluation over the specified epochs. Each model is monitored closely throughout the training process to track the progression of model loss functions. Initial observations indicate that models do not exhibit signs of overfitting, and the loss functions (DFL loss and box regression loss) reliably converge to low values, reflecting effective training practices.

Given that the dataset contains a single class (insulators), classification loss (Cls Loss) is not monitored, as it is not relevant in this context.

**Figure 13.**
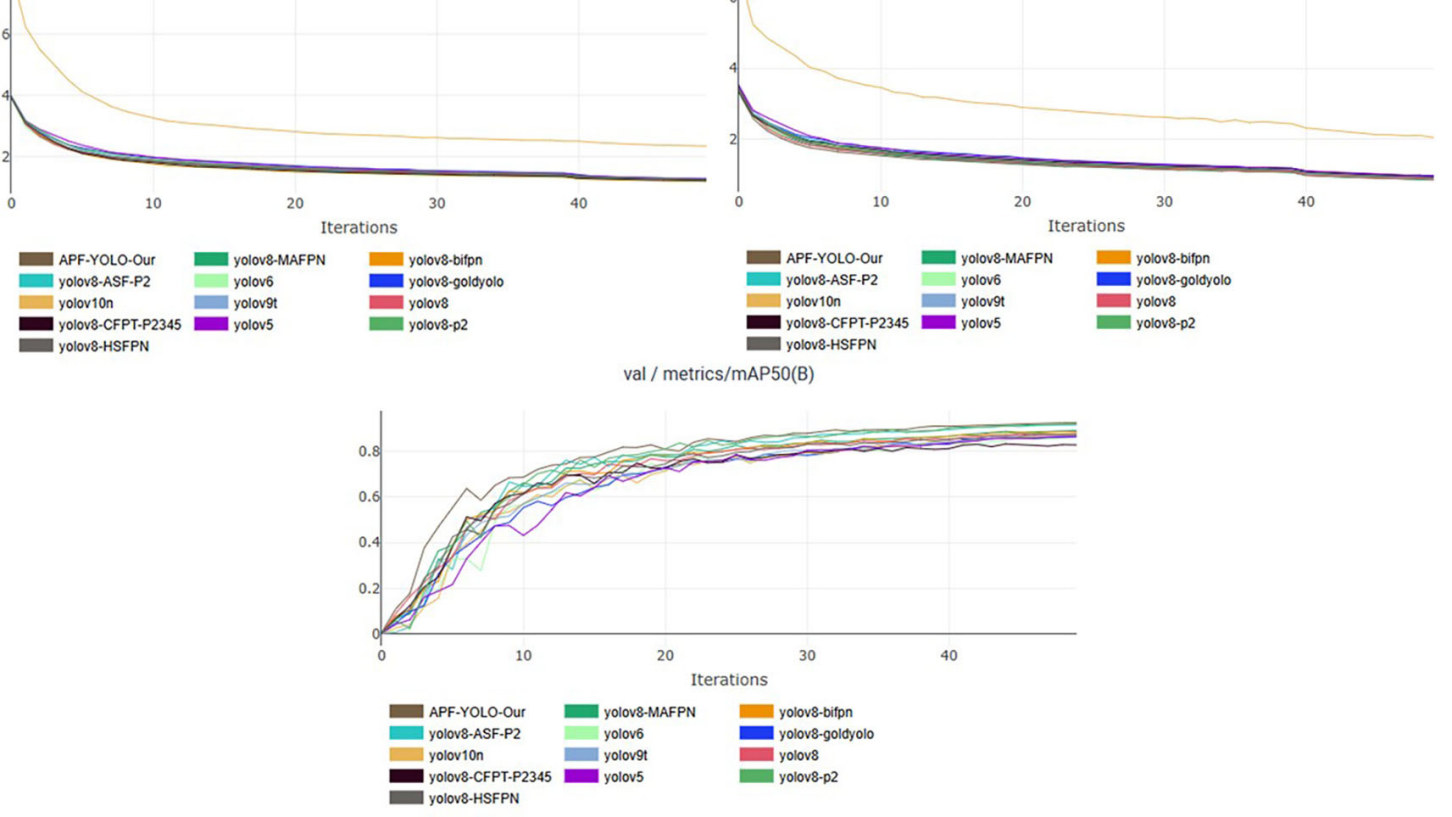
Training Stability and Validation Convergence Across YOLO Variants: Assessing Overfitting and Epoch Efficiency.

To visualize the training efficacy, performance curves are generated, demonstrating stability and convergence around the 50-epoch step. While we primarily focus on the mean Average Precision (mAP) metric to minimize clutter in results presentation, other evaluation metrics exhibit similar trends, reinforcing the good training phase. These findings are illustrated in the accompanying Figure 13, providing a clear picture of the training outcomes.

All hyperparameters used in this study can be found in the open-source code provided through this link: [ https://github.com/phd-benel/APF-YOLOv8].

## Results

### 1) Comparative analysis of our contribution against recent advancements

The current evaluation aims to analyze the performance of state-of-the-art (SOTA) models for insulator detection, a complex task involving multi-scale detection, small object sizes, and intra-class invariance. These challenges make it difficult for models to achieve exceptionally high performance (e.g., near 99% accuracy) across all metrics, as seen in the Table 4. The analysis covers key metrics such as mAP 50, mAP 50-95, precision, recall, and computational efficiency, shedding light on how each model balances detection performance with computational cost.

**Table 4.** A comparative study on various metrics across validation set.

Models	MaP@0.5	MaP@0.5:0.9	Precision	Recall	Gflops	Inference time (in ms)	Parameters (in millions M)
yolov8n_baseline	0.876	0.6365	0.8938	0.8111	8.194	2.694	3.01 M
yolov8n-p2	0.9173	0.6621	**0.9273**	0.8456	12.358	3.866	2.92 M
yolov9t	0.866	0.6317	0.8899	0.784	7.849	3.119	2.00 M
yolov8n-MAFPN	0.8893	0.6474	0.8941	0.8233	8.862	2.394	2.99 M
yolov8n-HSFPN	0.8731	0.6267	0.8922	0.7881	**6.945**	2.208	1.93 M
Yolov8n-goldyolo	0.8622	0.6125	0.9074	0.782	10.512	7.787	6.02 M
yolov8n-CFPT-P2345	0.8265	0.5997	0.8046	0.8054	9.62	14.279	**1.72 M**
yolov8n-bifpn	0.8839	0.6406	0.8755	0.8233	7.24	2.227	1.99 M
yolov8n-ASF-P2	0.9149	0.6574	0.8918	**0.8549**	12.138	4.516	2.49 M
yolov6n	0.8582	0.6206	0.8941	0.7929	11.869	3.931	4.24 M
yolov5n	0.8646	0.6101	0.8714	0.7918	7.177	**1.933**	2.50 M
yolov10n	0.87	0.6182	0.8785	0.7881	8.393	3.186	2.71 M
APF-YOLO -Our	**0.9241**	**0.6801**	**0.9243**	**0.8561**	12.779	5.163	3.57 M

The bolded values represent the best results for their respective metrics.

Overview of Model Performance

The results indicate that APF-YOLO achieves the best overall performance, with a mAP 50 of 0.9241, mAP 50-95 of 0.6801, precision of 0.9243, and recall of 0.8561. These results highlight its effectiveness in detecting insulators with diverse scales and intra-class variations. However, these gains come with increased computational requirements, as indicated by 12.779 GFLOPs, 3.6 million parameters, and an inference speed of 5.163 ms. Despite the additional computational demands, APF-YOLO demonstrates an optimal balance between accuracy and resource consumption, making it suitable for multi-scale detection and complex object variations.

Analysis with Specific Metrics

Compared to yolov8n_baseline, which serves as a standard benchmark, APF-YOLO achieves a 5.5% increase in mAP 50 (0.9241 vs. 0.8760) and a 6.9% increase in mAP 50-95 (0.6801 vs. 0.6365). It also achieves a 3.4% improvement in precision (0.9243 vs. 0.8938) and a 5.5% improvement in recall (0.8561 vs. 0.8111). These gains suggest that APF-YOLO effectively addresses the complexities of detecting insulators across multiple scales and types. However, this comes at the cost of 56% higher GFLOPs, 18.7% more parameters, and a 92% slower inference speed compared to yolov8n_baseline. This trade-off indicates that while accuracy significantly improves, computational efficiency decreases, highlighting a challenge for real-time applications.

Comparative Analysis Against Competitors

When compared to yolov8n-p2, APF-YOLO achieves a 0.74% higher mAP 50 (0.9241 vs. 0.9173) and a 2.72% increase in mAP 50-95 (0.6801 vs. 0.6621). While precision is slightly lower (0.9243 vs. 0.9273), APF-YOLO achieves 1.23% higher recall (0.8561 vs. 0.8456), suggesting better overall coverage of detected objects. The computational cost is also slightly higher, with 3.4% more GFLOPs, 22% more parameters, and 33% slower inference speed. Despite this, APF-YOLO achieves better generalization and recall, making it more suitable for comprehensive detection tasks.

Other competitors like Yolov10n, Yolov8n-MAFPN, Yolov8n-HSFPN, and Yolov8n-goldyolo show varied performance levels. For instance, Yolov10n achieves a 6.2% lower mAP 50 (0.9241 vs. 0.8700) and a 9.1% lower mAP 50-95 (0.6801 vs. 0.6182). Similarly, Yolov8n-HSFPN has 5.8% lower mAP 50 (0.8731) and 7.9% lower mAP 50-95 (0.6267), along with a 3.6% lower precision. Meanwhile, Yolov8n-MAFPN and Yolov8n-goldyolo also record lower performance in both mAP metrics and precision, with Yolov8n-MAFPN lagging behind by 3.9% in mAP 50 and 4.8% in mAP 50-95, and Yolov8n-goldyolo showing 6.2% lower mAP 50 and 10.3% lower mAP 50-95. These results underscore Our model’s competitive advantage in terms of accuracy, albeit with a higher computational cost.

**Figure 14.**
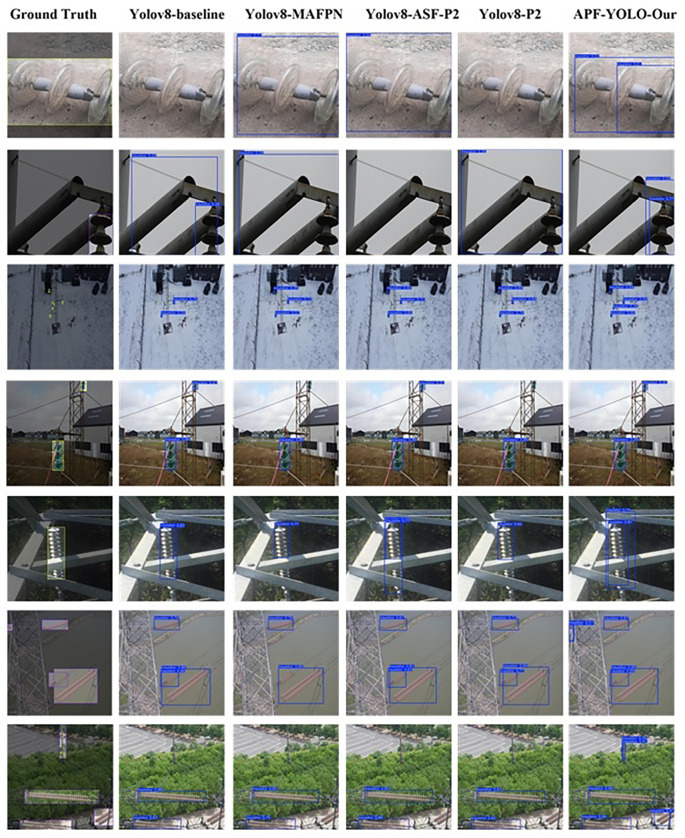
Illustrative Comparison of Model Predictions on the Validation Set. The results are displayed with bounding boxes, where the colors are arbitrary and do not convey any specific meaning. The ground truth represents the actual location of the object, serving as a reference for evaluation.

The results depicted in Figure 14 illustrate various challenging scenarios and highlight instances where APF-YOLO demonstrates strong performance compared to other models. In the first two rows, APF-YOLO shows precise bounding box placement for insulators of different types (glass in the first row and composite in the second), maintaining tight and accurate localization. Competing models, such as YOLOv8-baseline and YOLOv8-ASF-P2, either fail to detect these insulators entirely or produce overly large bounding boxes, which could limit their practical effectiveness.

In the third row, showing insulators captured from a distant perspective, APF-YOLO detects four insulators compared to three detected by YOLOv8-p2, indicating its potential for superior handling of distant objects. While no model detects all insulators in this scenario, APF-YOLO shows an advantage in managing this challenging case.

The fourth and fifth rows highlight the model’s ability to detect partially occluded insulators behind metallic pylons. APF-YOLO accurately identifies these occluded objects in their entirety, a critical feature for real-world deployments. Competitors like YOLOv8-p2 can’t manage this occlusion detection, further demonstrating the limitations of these alternative approaches.

In the sixth and seventh rows, APF-YOLO stands out in particularly extreme scenarios. For example, in row six, it successfully detects a heavily occluded insulator in the top-left corner, a scenario where other models struggle. Similarly, in row seven, APF-YOLO identifies an insulator (in the top) within a complex background, a task other models fail to achieve. These cases underscore its ability to handle extreme conditions, which are critical for UAV-based power line inspections.

Overall, Figure 14 illustrates that APF-YOLO performs effectively in a range of scenarios, including multiscale detection, occlusions, and challenging backgrounds. While the results do not suggest that the model is universally superior in all cases, they demonstrate its capability to handle extreme conditions—a key requirement for practical deployment in real-world inspection tasks.

Explanation of Robustness or Weaknesses in Performance

APF-YOLO achieves superior results across key metrics such as mAP 50 (0.9241), mAP 50-95 (0.6801), precision (0.9243), and recall (0.8561), reflecting its robustness in addressing multi-scale detection, small object recognition, and intra-class variance among different insulator types, including glass, porcelain, and composite. These strong results can be attributed to the innovative design of the AFAM module, which integrates specialized paths for handling both global semantic information and small object features.

In the AFAM module, one of the key design elements is the pathway dedicated to global semantic contextual information. This pathway is crucial for managing intra-class variance, as the global shape of insulators tends to be similar (e.g., disk-like shapes), despite variations in material type or structure. The module starts with a depthwise convolution (DW) with a kernel size of 11, which captures extensive contextual information across larger receptive fields. This operation is particularly effective for extracting global semantic features that help the model generalize across different insulator types. The large kernel size aligns well with detecting larger object sizes, which are generally found in deeper network layers.

Following the DW-11, the module incorporates Efficient Channel Attention (ECA) to refine channel-wise features. The deeper layers of the network contain a greater number of channels, making ECA especially effective in preserving relevant features and suppressing noise. By applying ECA after global semantic extraction, the model optimizes feature representation, ensuring that the most significant channels contribute to the detection of both large objects and intra-class variations.

The next component in the global pathway is the Context Anchor Attention (CAA). By leveraging vertical and horizontal convolutions, CAA enhances the model’s understanding of the spatial context. This mechanism further improves the model’s ability to differentiate between similar shapes by enriching global context representation, which is critical for accurately detecting insulators with varying sizes and orientations. The combination of these operations in the global pathway supports high precision and recall, demonstrating the model’s capability to capture complex spatial relationships and maintain generalization across different IoU thresholds. In parallel, the AFAM module also includes a pathway specifically designed for small object detection, addressing the challenges of multi-scale detection. This path starts with depthwise convolution (DW) with a kernel size of 3, which targets smaller receptive fields and is ideal for extracting fine-grained features. The use of a smaller kernel size is critical for detecting small objects, which often require precise localization. To enhance the detection of these smaller features, Efficient Local Attention (ELA) is applied, focusing on local spatial information while maintaining channel richness. This ensures accurate feature localization without compromising overall detection performance, contributing to Our model’s high mAP 50-95 and recall rates.

The architectural choices in APF-YOLO yield high detection performance, but they also introduce certain computational costs. The three-input processing of the AFAM module, along with the integration of large kernels, multiple attention mechanisms, and separate paths for global and local features, increases the computational load. This design contributes to the higher GFLOPs (12.779), a greater number of parameters (3.6 million), and a slower inference speed (5.163 ms), compared to conventional models that typically process a single input feature. The complexity of handling both global and local contexts simultaneously, while advantageous for accuracy, results in longer processing times and greater resource consumption.

In summary, APF-YOLO achieves the highest accuracy among the evaluated SOTA models, demonstrating superior performance in terms of mAP 50, mAP 50-95, and recall. It effectively balances precision and recall, making it a strong choice for insulator detection tasks that involve small objects and intra-class variations by incorporating specialized attention mechanisms and parallel paths for global and local feature extraction.

Future research could focus on optimizing APF-YOLO inference speed and reducing resource consumption. Strategies such as model pruning, quantization, knowledge distillation, and the use of efficient attention mechanisms could improve its applicability for real-time deployment in resource-constrained environments, while maintaining high accuracy across all metrics. Additionally, the model’s architecture makes it a promising candidate for scaling applications, such as cloud computing, where large-scale models can leverage increased computational capacity without compromising detection performance. While the computational costs are higher due to the complex architecture, the trade-off is justified by the model’s strong performance across all key metrics.

### 2) Ablation study

I. **Ablation Study: Evaluating Scaling in APF-YOLO Compared to Baseline Models YOLOv8 and YOLOv8-P2** ( Table 5)

**Table 5.** Scaling ability of models on validation set (from nano to small scall).

Model	mAP 50	mAP 50-95	Precision	Recall	GFLOPs	Parameters	Speed (ms)
Yolov8-P2-nano	0.9173	0.6621	0.9273	0.8456	12.358	2,926,692	3.866
Yolov8-P2-small	0.9330 (+0.0157)	0.6940 (+0.0319)	0.9300 (+0.0027)	0.8780 (+0.0324)	36.947 (+24.589)	10,637,236 **(+7,710,544)**	6.932 (+3.066)
Yolov8_baseline_nano	0.8760	0.6365	0.8938	0.8111	8.194	3,011,043	2.694
Yolov8_baseline_small	0.8937 (+0.0177)	0.6626 (+0.0261)	0.9174 **(+0.0236**)	0.8301 (+0.0190)	28.647 **(+20.453)**	11,135,987 (+8,124,944)	4.562 **(+1.868)**
APF-YOLO nano	0.9241	0.6801	0.9243	0.8561	12.779	3,572,454	5.163
APF-YOLO small	0.9456 ( **+0.0215**)	0.7176 ( **+0.0375**)	0.9469 (+0.0226)	0.8915 **(+0.0354)**	37.545 (+24.766)	12,894,266 (+9,321,812)	9.652 (+4.489)

The bolded values represent the best results for their respective metrics.

The primary goal of this ablation study is to evaluate how well our proposed model scales from nano to small configurations compared to baseline models, YOLOv8 and YOLOv8-P2. Scaling is a critical feature for models used in edge computing and UAV-based detection, as it enables adapting model complexity to different deployment scenarios while maintaining robust performance. By isolating the nano and small configurations in this study, we assess the effectiveness of APF-YOLO in leveraging additional computational resources and parameters to improve detection performance relative to baseline models.

The scaling performance of APF-YOLO is evident in the substantial gains observed in both mAP 50 and mAP 50-95 metrics when transitioning from nano to small configurations. For instance, APF-YOLO nano achieves a mAP 50 of 0.9241, which improves to 0.9456 in APF-YOLO small, a relative increase of 2.3%. This scaling gain is more pronounced than that observed in YOLOv8-P2, where mAP 50 increases from 0.9173 in the nano version to 0.9330 in the small version, reflecting a smaller relative improvement of 1.7%. Similarly, YOLOv8-baseline shows a smaller scaling improvement, with mAP 50 increasing from 0.8760 in the nano version to 0.8937 in the small version.

The mAP 50-95 metric, which evaluates detection robustness across varying IoU thresholds, follows a similar trend. APF-YOLO achieves a gain of 0.0375 (from 0.6801 to 0.7176) when scaled from nano to small, significantly outperforming YOLOv8-P2 (0.0319 gain) and YOLOv8-baseline (0.0261 gain). These results indicate that APF-YOLO effectively utilizes additional parameters and computational resources to generalize better across scales, demonstrating superior scalability in performance compared to baseline models.

Precision and recall metrics further highlight the advantages of APF-YOLO in scaling. In the nano configuration, APF-YOLO nano achieves a precision of 0.9243 and a recall of 0.8561. When scaled to APF-YOLO small, these metrics improve to 0.9469 (precision) and 0.8915 (recall), reflecting increases of 2.4% and 3.9%, respectively. These improvements are significantly greater than those observed in YOLOv8-P2, where precision increases by 0.3% and recall by 3.2%, and YOLOv8-baseline, which shows improvements of 2.4% in precision and 1.9% in recall. This suggests that APF-YOLO better leverages scaling to enhance detection reliability, particularly in scenarios with high intra-class variance or multiscale object detection challenges.

While scaling improves performance, it also incurs additional computational costs, as reflected in the GFLOPs and inference speed metrics. For APF-YOLO, GFLOPs increase from 12.779 in the nano configuration to 37.545 in the small configuration, a relative increase of 193%. Similarly, inference speed decreases from 5.163 ms to 9.652 ms, reflecting a 87% slowdown. These changes are comparable to the scaling costs in YOLOv8-P2, where GFLOPs increase from 12.358 to 36.947 (199%) and inference time increases from 3.866 ms to 6.932 ms (79%). YOLOv8-baseline shows similar trends, with GFLOPs increasing by 250% and inference speed decreasing by 69%.

Despite these computational increases, APF-YOLO small consistently outperforms baseline small models in terms of accuracy and robustness, indicating a more efficient utilization of additional resources. This efficiency is crucial for edge computing scenarios, where computational overhead must be justified by substantial gains in detection performance.

The ablation study clearly demonstrates that APF-YOLO scales more effectively than both YOLOv8 and YOLOv8-P2. While all models show performance improvements when scaled, the relative gains in mAP 50, mAP 50-95, precision, and recall are consistently higher in our model. Additionally, the computational cost and inference speed penalties incurred by scaling are comparable across all models, but the performance advantages of APF-YOLO small justify these trade-offs more effectively.

For instance, the mAP 50-95 gain of 0.0375 in APF-YOLO is significantly higher than the gains of 0.0319 and 0.0261 observed in YOLOv8-P2 and YOLOv8-baseline, respectively, even though all models experience similar increases in GFLOPs and decreases in speed. This highlights that APF-YOLO achieves a better balance between performance and computational efficiency, making it a more scalable and practical choice for UAV-based detection applications.

The results of this first ablation study underscore the superior scalability of APF-YOLO compared to YOLOv8 and YOLOv8-P2. By effectively leveraging additional computational resources and parameters, APF-YOLO small achieves the highest performance gains among all tested models, particularly in challenging detection scenarios involving multiscale objects and high intra-class variance. These findings validate the design choices in our architecture, demonstrating its robustness and adaptability for edge computing applications in UAV-based systems. Consequently, APF-YOLO sets a new benchmark for scalable, high-performance object detection in resource-constrained environments.

II. **Ablation Study: Optimal Placement of the AFAM Module in the Neck** ( Table 6)

**Table 6.** Investigation into the impact of afam placement in the neck.

AFAM Placement	mAP@0.5	mAP@0.5:0.95	Precision	Recall	GFLOPs	Parameters (in millions)	Speed (ms)
AFAM - Last	**0.9241**	0.6801	**0.9243**	0.8561	12.779	**3.572M**	3.866
AFAM - Beginning	0.9163	0.6636	0.9140	0.8599	**12.315**	**3.424M**	6.932 (+3.066)
AFAM - 2 Last + 2 Beginning	**0.9262**	**0.6876**	0.9168	**0.8715**	14.071	4.320M	2.694

The bolded values represent the best results for their respective metrics.

The AFAM module is an architectural component aimed at enhancing feature refinement in object detection models. Its placement within the neck plays a crucial role in determining the balance between detection performance and computational efficiency. In this study, we evaluate three different placement strategies for the AFAM module—at the last stage of the neck, at the beginning of the neck, and distributed across both positions (two at the beginning and two at the last). The goal is to identify the optimal placement that maximizes detection performance while maintaining manageable computational costs and inference speed.

Performance Metrics Across Different Configurations

1. AFAM at the Last Stage
  Placing the AFAM module at the last stage achieves strong performance metrics, with a mAP 50 of 0.9241 and mAP 50-95 of 0.6801. This configuration delivers competitive precision (0.9243) and recall (0.8561), suggesting that the module effectively refines higher-level features for improved object detection. The computational cost is moderate, with 12.779 GFLOPs and 3.57M parameters, resulting in an inference speed of 5.163 ms. This placement balances accuracy and efficiency, making it suitable for scenarios where moderate computational overhead is acceptable.
2. AFAM at the Beginning of the Neck
  Placing the AFAM module at the beginning of the neck results in a slight decline in detection performance. The mAP 50 drops to 0.9163, and mAP 50-95 decreases to 0.6636. Precision also declines to 0.9140, while recall slightly increases to 0.8599. The lower precision indicates that the module struggles to fully exploit the early-stage features for object refinement. This configuration has the lowest computational cost, with 12.315 GFLOPs and 3.42M parameters, and maintains an inference speed of 5.171 ms. While it is computationally efficient, its reduction in accuracy may limit its applicability for tasks requiring high detection precision.
3. Distributed Placement (Two at the Beginning and Two at the Last)
  The distributed placement achieves the best overall performance among the three configurations. It records the highest mAP 50 (0.9262) and mAP 50-95 (0.6876), indicating superior detection accuracy and robustness across IoU thresholds. Precision (0.9168) and recall (0.8715) also reach their highest levels, reflecting the ability of this configuration to capture both fine-grained details and high-level semantic features. However, this configuration incurs the highest computational cost, with 14.071 GFLOPs and 4.32M parameters, resulting in a slower inference speed of 6.509 ms. Despite the increase in resource demands, the performance gains justify the trade-offs, making this configuration ideal for scenarios prioritizing accuracy over speed.

Computational Cost and Efficiency Analysis

The placement of the AFAM module significantly affects the model’s computational efficiency. Placing the module at the beginning minimizes computational costs (12.315 GFLOPs) and keeps inference speed low (5.171 ms), making it the most resource-efficient configuration. However, the performance trade-offs in terms of mAP 50 and precision may not be suitable for applications requiring high detection accuracy.

In contrast, placing the module at the last stage slightly increases computational cost (12.779 GFLOPs) while maintaining a similar inference speed (5.163 ms). This configuration strikes a balance between accuracy and efficiency, making it a practical choice for real-time applications where moderate computational overhead is acceptable.

The distributed placement introduces the highest computational cost (14.071 GFLOPs) and slows inference speed to 6.509 ms. While the additional overhead limits its suitability for resource-constrained environments, the substantial performance improvements in detection metrics make it an attractive choice for accuracy-critical tasks.

The ablation experiments reveal that the distributed placement of the AFAM module—two at the beginning and two at the last stage of the neck—yields the best overall performance. This configuration maximizes detection accuracy (mAP 50: 0.9262, mAP 50-95: 0.6876) and robustness while maintaining competitive precision and recall. Although it incurs higher computational costs and slower inference speeds, the performance gains justify its application in scenarios where accuracy is paramount. For applications prioritizing computational efficiency and real-time processing, the AFAM module’s placement at the last stage provides a viable alternative, balancing accuracy and resource demands. These insights demonstrate the critical impact of architectural decisions on model performance and their implications for edge computing deployments.

III. **Ablation Study: Evaluating the Impact of Module Placement on the AFAM Module** ( Table 7)

**Table 7.** Comparison of various module placements in afam module.

Module Placement in AFAM	mAP@0.5	mAP@0.5:0.95	Precision	Recall	GFLOPs	Parameters (in millions)	Speed (ms)
Only Concat Feature Alignment	0.9113	0.6566	0.9131	0.8497	**12.158**	**3.254M**	3.846
No DW + Attention	**0.9237**	0.6722	0.9194	0.8526	12.624	3.572M	4.598
DW + No Attention	**0.9250**	0.6740	0.9103	**0.8691**	12.525	3.572M	6.932 (+3.066)
No Small Local Path	0.9196	0.6760	0.9124	0.8619	12.768	3.572M	2.694
No Large Context Path	0.9188	0.6687	0.9158	0.8586	12.381	3.572M	3.879
APF-YOLO(All)	**0.9241**	**0.6801**	**0.9243**	0.8561	12.779	3.57 M	5.163

The bolded values represent the best results for their respective metrics.

This ablation study investigates the effect of various module placements and configurations within the AFAM module of the APF-YOLO model. Specifically, we explore the role of depthwise convolutions (DW), attention mechanisms, and the inclusion of local and global pathways in enhancing the model’s detection performance. By systematically altering these components, we aim to determine their contribution to the AFAM module’s overall effectiveness.

1. No Depthwise Convolutions with Attention
  This configuration excludes depthwise convolutions while retaining attention mechanisms. The model achieves a mAP 50 of 0.9237 and a mAP 50-95 of 0.6722. Precision is recorded at 0.9194, and recall is 0.8526. With 12.624 GFLOPs and 3.57M parameters, the inference speed is 4.598 ms. While the model retains good performance, the lack of depthwise convolutions reduces its ability to extract fine-grained spatial features, leading to slightly lower recall.
2. Depthwise Convolutions Without Attention
  Introducing depthwise convolutions while excluding attention mechanisms slightly improves performance, with mAP 50 increasing to 0.9250 and mAP 50-95 to 0.6740. Recall rises to 0.8691, indicating better coverage of true positives, but precision drops to 0.9103, reflecting a trade-off in the model’s ability to minimize false positives. This configuration has the lowest computational cost, requiring 12.525 GFLOPs and maintaining a similar parameter count and inference speed (4.6 ms).
3. No Enhancements (Only Concatenation of Features)
  A baseline configuration with only feature concatenation achieves the lowest performance across all metrics, with a mAP 50 of 0.9113 and mAP 50-95 of 0.6566. Precision is 0.9131, and recall is 0.8497. The computational cost is the lowest, with 12.158 GFLOPs, 3.25M parameters, and a speed of 3.846 ms. These results demonstrate that the AFAM module’s advanced mechanisms are essential for achieving high detection accuracy.
4. No Small Local Path
  Excluding the small local path reduces the model’s ability to capture fine-grained details, particularly for small objects. The mAP 50 drops to 0.9196, and mAP 50-95 is 0.6760. Precision is 0.9124, and recall is 0.8619. With 12.768 GFLOPs and a speed of 4.958 ms, this configuration demonstrates that the small local path contributes significantly to the model’s ability to detect small objects.
5. No Large Context Path
  Removing the large context path affects the model’s ability to capture global contextual information, resulting in a mAP 50 of 0.9188 and a mAP 50-95 of 0.6687. Precision is 0.9158, and recall is 0.8586. This configuration requires 12.381 GFLOPs, 3.57M parameters, and achieves a speed of 3.879 ms. The results highlight that the large context path is essential for robust detection of larger objects and for managing intra-class variance.

Comparative Analysis of Configurations

The results indicate that the AFAM module benefits most from configurations that integrate both depthwise convolutions and attention mechanisms. Specifically, the combination of depthwise convolutions and attention mechanisms achieves the best balance between performance metrics and computational efficiency. The following observations stand out:

Best Performance: The configuration with depthwise convolutions and no attention achieves the highest recall (0.8691), making it suitable for applications requiring high coverage of true positives. However, it sacrifices precision, which could lead to more false positives.

Balanced Trade-Off: The configuration with both depthwise convolutions and attention mechanisms achieves a strong balance between precision (0.9194) and recall (0.8526). It demonstrates the effectiveness of combining local feature extraction (via depthwise convolutions) and feature weighting (via attention mechanisms).

Small and Large Path Contributions: Excluding either the small local path or the large context path reduces performance, indicating their complementary roles in handling small and large objects, respectively. The small local path is critical for capturing fine-grained details, while the large context path enhances the model’s ability to interpret global spatial relationships.

The ablation study underscores the importance of combining depthwise convolutions and attention mechanisms within the AFAM module. The inclusion of both small and large pathways is essential for achieving robust multiscale detection. While configurations with depthwise convolutions alone are computationally efficient, the integration of attention mechanisms further refines feature representations, leading to improved detection accuracy and robustness.

For optimal performance in real-world UAV-based detection applications, the full AFAM module—integrating both pathways and leveraging advanced mechanisms—emerges as the most effective design choice.

## Discussion

While APF-YOLO achieves significant advancements in handling multiscale detection and intra-class variance, several limitations must be addressed for broader applicability, particularly in resource-constrained environments. The additional computational demands introduced by its enhanced neck design, including advanced attention mechanisms and multi-path fusion modules, result in increased inference times and model complexity. However, these computational requirements remain within acceptable bounds for real-time performance in server-grade configurations, achieving inference times below the critical threshold of 33.3 ms per frame, corresponding to approximately 30 frames per second (fps).

Nevertheless, deploying APF-YOLO on edge devices, such as NVIDIA Jetson or Google Coral Edge TPU, which are commonly used in UAVs and embedded systems, remains a challenge. Edge devices typically have limited computational resources, memory, and power capabilities, which may constrain the feasibility of deploying models with APF-YOLO’s complexity. Although direct edge processing is not the main focus of this research, YOLOv8—the foundation of our model—has already demonstrated efficient real-time performance on edge devices. ^46^^,^^47^ For example, YOLOv8-nano optimized with TensorRT achieves around 100 FPS on a Jetson Orin Nano, confirming its practical suitability for resource-constrained deployments. ^48^ Given that our model is built upon the YOLOv8 architecture, we hypothesize that APF-YOLOV8 would remain within the same operational category in terms of deployment feasibility.

Future work should include extensive testing on edge hardware to evaluate the model’s practical applicability in such environments. Additionally, optimization techniques such as pruning, quantization, and knowledge distillation should be explored to reduce the model’s size and computational demands while maintaining its detection capabilities. A promising direction could involve integrating lightweight architectures, such as MobileNet, ^49^ as the backbone feature extractor, enabling a faster and more energy-efficient inference pipeline while retaining the accuracy benefits provided by APF-YOLO’s neck design. Futhermore, future work could also explore adaptive inference techniques, such as focusing on critical regions within the image or batching strategies for efficient frame processing. Additionally, leveraging hardware accelerators specifically designed for deep learning, like NVIDIA TensorRT or Google Coral Edge TPU, could further optimize the model’s latency without compromising its detection accuracy.

Another notable limitation stems from the scope and diversity of the dataset used for training and evaluation. While the Merged Public Insulator Dataset (MPID) enhances the availability of data for insulator detection by consolidating images from various sources and scenarios, it remains inherently limited in capturing the full complexity of real-world UAV-based inspection tasks. Factors such as extreme occlusion, variable lighting conditions, weather challenges (e.g., fog, rain, or snow), and diverse geographical backgrounds pose significant challenges that require further investigation.

In particular, edge cases—such as insulators that are heavily occluded or captured from distant viewpoints—continue to pose difficulties for APF-YOLO. Although the model performs robustly under controlled or moderately challenging conditions, its precision in extreme scenarios involving partial visibility or long-range detection is not yet fully reliable. This is a common limitation shared by many object detection models, as no architecture can guarantee optimal performance across all possible real-world conditions.

To address these challenges, we propose several promising directions for future research. First, we intend to explore specialized frameworks designed for small and distant object detection, such as SOD-YOLO, ^50^ which leverages a dual-stream attention-guided mechanism and a lightweight head to enhance detection accuracy without significantly increasing computational cost. Second, we plan to investigate occlusion-aware data augmentation techniques, including Hide-and-Seek, ^51^ which randomly occlude regions of the image during training. This strategy encourages the model to learn from contextual information, improving its generalization under partial visibility.

These enhancements—combined with broader dataset diversification and extensive testing under real UAV flight conditions—will be essential to ensure operational robustness and generalization in real-world deployments. While the present study demonstrates strong performance in structured settings, future work will focus on validating the model’s reliability under more dynamic and unpredictable field conditions.

Additionally, the generalizability of APF-YOLO to domains beyond power line inspections has not been thoroughly explored. While its modular architecture and design innovations suggest potential adaptability to tasks such as industrial safety monitoring, agricultural object detection, or autonomous navigation, empirical validation across these domains remains an open question. Incorporating domain adaptation techniques or transfer learning approaches could enhance the model’s ability to adapt to new tasks with minimal additional training data, broadening its applicability and impact.

In summary, while APF-YOLO shows significant promise for improving small object detection and handling intra-class variance in UAV-based power line inspections, several areas require further attention. Addressing computational efficiency, extending dataset diversity, validating real-world performance, and exploring cross-domain adaptability are crucial for enhancing its scalability and operational deployment. By focusing on these aspects, APF-YOLO can evolve into a more versatile and practical solution for diverse object detection challenges in complex environments.

## Conclusion

This study introduces APF-YOLO, a refined YOLOv8-based model tailored to the challenges of UAV-based power line inspections, particularly multiscale object detection and intra-class variance. By integrating the Adaptive Path Fusion (APF) neck and incorporating an Adaptive Feature Alignment Module (AFAM) in the neck, alongside advanced attention mechanisms such as Efficient Local Attention (ELA), Efficient Channel Attention (ECA), and Context Anchor Attention (CAA), APF-YOLO aims to enhance detection precision across varying object sizes and effectively differentiate between visually similar insulators, such as glass, porcelain, and composite types. These innovations enable the model to harmonize spatial detail and semantic understanding, addressing the complexities of real-world operational conditions.

Experimental results demonstrate that APF-YOLO consistently surpasses competing YOLO neck configurations in key performance metrics. The model achieves at least +0.74% improvement in mAP@0.5, +2.71% in mAP@0.5:0.9, and +1.24% in recall over other YOLO-based models, showcasing its enhanced detection capabilities across a broad spectrum of scenarios. Additionally, APF-YOLO maintains an inference time within the threshold required for real-time applications (under 33.3 ms in server-grade configurations), ensuring its practicality for UAV-based inspection workflows. The development of the Merged Public Insulator Dataset (MPID) further supports this work, providing a diverse and comprehensive benchmark for evaluating insulator detection models, contributing significantly to the research community.

While APF-YOLO exhibits substantial advancements, the study acknowledges several limitations. The model’s computational demands, although manageable in server-grade environments, may pose challenges for edge device deployment, such as on UAVs with constrained hardware resources. Future work will explore optimization techniques like pruning, quantization, and knowledge distillation to make APF-YOLO suitable for resource-limited platforms. Lightweight alternatives, such as using MobileNet as a feature extractor, and hardware-specific accelerations, like NVIDIA TensorRT, will also be investigated to improve efficiency without sacrificing performance. Moreover, while MPID broadens dataset availability, it remains limited in simulating real-world dynamic conditions. Testing APF-YOLO in live UAV operations and validating it across diverse datasets and domains—such as autonomous navigation or agricultural monitoring—are critical next steps.

Looking forward, future research will prioritize enhancing APF-YOLO’s adaptability for edge computing, further refining its architecture to meet real-world deployment needs, and leveraging domain adaptation techniques to improve robustness across diverse operational conditions. Expanding dataset diversity and exploring hybrid architectures will also be key to advancing APF-YOLO’s versatility.

In conclusion, APF-YOLO marks a significant step forward in leveraging UAVs for infrastructure monitoring, offering a promising solution for multiscale detection and intra-class variance handling. While challenges remain, the model hopes to establish a solid foundation for further innovations in UAV-based object detection, paving the way for safer and more efficient power line inspections and related applications.

## Ethics and consent

Ethical approval and consent were not required.

## Data Availability

The underlying data supporting the findings of this study have been made openly available in accordance with the FAIR (Findable, Accessible, Interoperable, and Reusable) and open data policy. The dataset, titled “MPID: An Open-Source Insulator Dataset for UAV Inspection of Power Lines”, is accessible through the Zenodo repository: MPID: An Open-Source Insulator Dataset for UAV Inspection of Power Lines DOI: 10.5281/zenodo.14604384 ^52^ License and access: The dataset is available under the terms of the Creative Commons Attribution 4.0 International license (CC-BY 4.0). This license permits unrestricted use, distribution, and reproduction in any medium, provided the original authors and source are credited. De-identification: The dataset does not involve human participants or personal data. It comprises publicly available aerial imagery of insulators, and all data were de-identified at the source. No sensitive or personally identifiable information is included. Repository details: The Zenodo repository ensures data stability and long-term preservation. Metadata has been formatted according to repository-specific guidelines to facilitate discoverability and interoperability. Usage and citation: Researchers are encouraged to reuse and build upon the dataset while appropriately citing this publication and the dataset using the following citation format.

## Software availability

• Source code available from: https://github.com/phd-benel/APF-YOLOv8
• Archived software available from: https://zenodo.org/records/14604484
• License: Software is available under the terms of the Creative Commons Attribution 4.0 International (CC-BY 4.0) license.
