# Supplementary material for: APF-YOLOV8: Enhancing Multiscale Detection and Intra-Class Variance Handling for UAV-Based Insulator Power Line Inspections
Source: F1000Res. 2025 Jun 23;14:141. Originally published 2025 Jan 28. [Version 2] doi: 10.12688/f1000research.160650.2 (PMC12391864; doi:10.12688/f1000research.160650.2)
Supplement: Peer review history

# Reviewer report: Reviewer response for version 2

**DOI:** 10.5256/f1000research.183851.r394066

**Published:** 2025-08-27

**Authors:** Md Sabid Hasan

**Affiliations:** 1 Soil and Water Systems, University of Idaho College of Agricultural and Life Sciences, Idaho, Idaho, USA

The revised version is looking good for indexing. Minor details are well written. You can add the following paper to the references(refer to 1)

Is the work clearly and accurately presented and does it cite the current literature?

Partly

If applicable, is the statistical analysis and its interpretation appropriate?

Partly

Are all the source data underlying the results available to ensure full reproducibility?

Yes

Is the study design appropriate and is the work technically sound?

Yes

Are the conclusions drawn adequately supported by the results?

Yes

Are sufficient details of methods and analysis provided to allow replication by others?

Yes

Reviewer Expertise:

Intelligent Transportation Systems, Deep Learning, Reinforcement Learning, Object Detection and Classification, Computer Vision, Image Classification, Transfer Learning, Machine Learning, Robot Navigation, Multi-robot Task Allocation, Remote Sensing, Power Plant Operation, Power Transmission system, Precision Agriculture.

I confirm that I have read this submission and believe that I have an appropriate level of expertise to confirm that it is of an acceptable scientific standard.

## References

[1] : Enhancing the reliability of power grids: A YOLO based approach for insulator defect detection. *e-Prime - Advances in Electrical Engineering, Electronics and Energy* .2024;9: 10.1016/j.prime.2024.100663 10.1016/j.prime.2024.100663

# Reviewer report: Reviewer response for version 2

**DOI:** 10.5256/f1000research.183851.r394065

**Published:** 2025-06-26

**Authors:** Jeya prakash Kadambarajan

**Affiliations:** 1 Kalasalingam Academy of Research and Education, Krishnankoil, Tamil Nadu, India

Approved.

Is the work clearly and accurately presented and does it cite the current literature?

Yes

If applicable, is the statistical analysis and its interpretation appropriate?

Yes

Are all the source data underlying the results available to ensure full reproducibility?

Yes

Is the study design appropriate and is the work technically sound?

Yes

Are the conclusions drawn adequately supported by the results?

Yes

Are sufficient details of methods and analysis provided to allow replication by others?

Yes

Reviewer Expertise:

Optimization algorithms, AI/ML Models, VLSI Physical Design Algorithms, Smarty Systems

I confirm that I have read this submission and believe that I have an appropriate level of expertise to confirm that it is of an acceptable scientific standard.

# Reviewer report: Reviewer response for version 1

**DOI:** 10.5256/f1000research.176578.r369717

**Published:** 2025-05-12

**Authors:** Md Sabid Hasan, Md Mostafizur Rahman Komol

**Affiliations:** 1 Soil and Water Systems, University of Idaho College of Agricultural and Life Sciences, Idaho, Idaho, USA; 2 Electrical Engineering and Robotics, Queensland University of Technology Centre for Robotics, Brisbane, Queensland, Australia

The research paper presents APF-YOLO, an enhanced variant of the YOLOv8 model, designed specifically to address significant challenges encountered in UAV-based power line inspections, particularly multiscale object detection and intra-class variance handling. Insulator detection is complicated by scale variations due to UAV altitude fluctuations and visual similarities among different insulator types, such as glass, porcelain, and composite. To tackle these challenges, the APF-YOLO model integrates an innovative Adaptive Path Fusion (APF) neck combined with an Adaptive Feature Alignment Module (AFAM).

 These modules utilize advanced attention mechanisms, including Efficient Local Attention (ELA), Efficient Channel Attention (ECA), and Context Anchor Attention (CAA), enhancing the fusion of fine-grained spatial details and broader semantic context.

 A major contribution of the study is the creation of the Merged Public Insulator Dataset (MPID), composed of diverse imagery capturing various real-world conditions, such as different lighting conditions, distances, occlusions, and environmental settings. This comprehensive dataset significantly improves the generalizability and robustness of detection algorithms for real-world operational scenarios.

 Experimental results indicate APF-YOLO achieves superior performance, notably demonstrating approximately a 2.71% improvement in mAP@0.5:0.9 and a 1.24% increase in recall over state-of-the-art YOLO-based models. The authors benchmarked APF-YOLO against several advanced YOLO variants, providing extensive comparative evaluations that validate the efficacy of the proposed architectural modifications.

 However, the enhanced performance of APF-YOLO is accompanied by increased computational complexity, reflected by higher GFLOPs and extended inference times. This increased computational demand poses challenges for real-time applications on resource-constrained devices or edge computing platforms. Furthermore, while APF-YOLO significantly improves detection accuracy, it continues to struggle with precise detection of insulators under extreme occlusion and at distant viewpoints, suggesting the need for further refinements such as model pruning, lightweight feature extraction methods, or advanced data augmentation strategies. I have attached some papers you may add as your references.

Is the work clearly and accurately presented and does it cite the current literature?

Partly

If applicable, is the statistical analysis and its interpretation appropriate?

Partly

Are all the source data underlying the results available to ensure full reproducibility?

Yes

Is the study design appropriate and is the work technically sound?

Yes

Are the conclusions drawn adequately supported by the results?

Yes

Are sufficient details of methods and analysis provided to allow replication by others?

Yes

Reviewer Expertise:

Intelligent Transportation Systems, Deep Learning, Reinforcement Learning, Object Detection and Classification, Computer Vision, Image Classification, Transfer Learning, Machine Learning, Robot Navigation, Multi-robot Task Allocation, Remote Sensing, Power Plant Operation, Power Transmission system, Precision Agriculture.

We confirm that we have read this submission and believe that we have an appropriate level of expertise to confirm that it is of an acceptable scientific standard, however we have significant reservations, as outlined above.

## References

[1] : Enhancing the reliability of power grids: A YOLO based approach for insulator defect detection. *e-Prime - Advances in Electrical Engineering, Electronics and Energy* .2024;9: 10.1016/j.prime.2024.100663 10.1016/j.prime.2024.100663

[2] : Prediction of Drivers’ Red-Light Running Behaviour in Connected Vehicle Environments Using Deep Recurrent Neural Networks. *Machine Learning and Knowledge Extraction* .2024;6(4) : 10.3390/make6040136 2855-2875 10.3390/make6040136

[3] : Enhanced encapsulation efficiency and stability of aronia anthocyanins by encapsulation: A focus on ionic strength of wall materials. *Food Sci Biotechnol* .2025;34(10) : 10.1007/s10068-025-01875-2 2197-2206 10.1007/s10068-025-01875-2 40351731 PMC12064540

[4] M. S. Hasan, F. Fahim, A. A. Farabi and F. Rahman, "A Comparison between YOLOV7 and YOLOV5-based detection of Combat Targets and Camouflaged Militia," 2022 4th International Conference on Sustainable Technologies for Industry 4.0 (STI), Dhaka, Bangladesh, 2022, pp. 1-6, doi: 10.1109/STI56238.2022.10103281.

[5] : Fucoidan JHCF4s from Hizikia fusiformis against ethanol-induced damage in vitro and in vivo. *Food Sci Biotechnol* .2025;34(10) : 10.1007/s10068-025-01865-4 2295-2306 10.1007/s10068-025-01865-4 40351724 PMC12064512

[6] : Deep Transfer Learning Based Intersection Trajectory Movement Classification for Big Connected Vehicle Data. *IEEE Access* .2021;9: 10.1109/ACCESS.2021.3119600 141830-141842 10.1109/ACCESS.2021.3119600

# Reviewer report: Reviewer response for version 1

**DOI:** 10.5256/f1000research.176578.r378717

**Published:** 2025-05-06

**Authors:** Jeya prakash Kadambarajan

**Affiliations:** 1 Kalasalingam Academy of Research and Education, Krishnankoil, Tamil Nadu, India

Improve clarity and visibility of figures, ensuring all referenced diagrams and tables are present and legible. The conclusions are well-supported by results. The proposed neck modification is practical for UAV-based inspections where resource efficiency matters. APF-YOLO appears to be a solid step forward in object detection for high-variability visual data. The interpretation of results is strong, with clear evidence supporting APF-YOLO’s superior accuracy and recall at the expense of some computational efficiency.

Is the work clearly and accurately presented and does it cite the current literature?

Yes

If applicable, is the statistical analysis and its interpretation appropriate?

Yes

Are all the source data underlying the results available to ensure full reproducibility?

Yes

Is the study design appropriate and is the work technically sound?

Yes

Are the conclusions drawn adequately supported by the results?

Yes

Are sufficient details of methods and analysis provided to allow replication by others?

Yes

Reviewer Expertise:

Optimization algorithms, AI/ML Models, VLSI Physical Design Algorithms, Smarty Systems

I confirm that I have read this submission and believe that I have an appropriate level of expertise to confirm that it is of an acceptable scientific standard.
